# Base mediated spirocyclization of quinazoline: one-step synthesis of spiro-isoindolinone dihydroquinazolinones†

**DOI:** 10.1039/c9ra09567e

**Published:** 2020-03-04

**Authors:** Rapolu Venkateshwarlu, V. Narayana Murthy, Krishnaji Tadiparthi, Satish P. Nikumbh, Rajesh Jinkala, Vidavalur Siddaiah, M. V. Madhu babu, Hindupur Rama Mohan, Akula Raghunadh

**Affiliations:** Technology Development Centre, Custom Pharmaceutical Services, Dr. Reddy's Laboratories Ltd. Hyderabad 500049 India; Department of Organic Chemistry and FDW, Andhra University Visakhapatnam 530045 India raghunadha@drreddys.com; Department of Chemistry, CHRIST (Deemed to be University) Hosur Road Bengaluru 560029 India

## Abstract

A novel approach for the spiro-isoindolinone dihydroquinazolinones has been demonstrated from 2-aminobenzamide and 2-cyanomethyl benzoate in the presence of KHMDS as a base to get moderate yields. The reaction has been screened in various bases followed by solvents and a gram scale reaction has also been executed under the given conditions. Based on the controlled experiments a plausible reaction mechanism has been proposed. Further the substrate scope of this reaction has also been studied.

## Abstract 2

A novel approach for the spiro-isoindolinone dihydroquinazolinones has been demonstrated from 2-aminobenzamide and 2-cyanomethyl benzoate in the presence of KHMDS as a base to get moderate yields.

## Graphical abstract

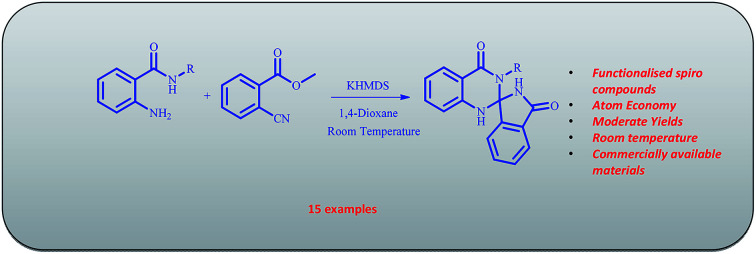

Because of their strong potent biologically activity, heterocyclic compounds have been a constant source of inspiration for the invention of new drugs especially for pharmaceutical and agro chemical industries.^1^ Indeed, investigation of novel methods for the synthesis of various natural products and heterocycles has always been a challenging task in modern organic chemistry. Amid all, spiro based scaffolds have been found to be very interesting because of their structural diversity. In spite of their intrinsic structures and immense biological activity there is a tremendous demand for the chemistry of spiro-isoindolinone dihydroquinazolinones.^2^ Indeed, nitrogen containing heterocyclic compounds like spiro-oxindole, spiro-isoindoline, spiro-isoindolinone are playing a significant role in medicinal chemistry and synthetic transformations.^3^ Moreover these compounds present in many natural products as a core unit like Lennoxamine, Zopiclone, Taliscanine and Pazinaclone (Fig. 1). In addition, many unnatural spiro-isoindolinones show significant biological activities acting as anti HIV-1, antiviral, antileukemic, anesthetic and antihypertensive agents.^4,5^ Notably, the spiro-isoindolinone dihydroquinazolinone unit has been found to be a combination of two potent pharmacophore units of dihydroquinazolinone and spiro-isoindolinone. Inspite of their remarkable biological activity afore mentioned, various methods have been developed for their synthesis like lithiation approaches, base mediated protocols, Diels–Alder and Wittig reactions, electrophilic and radical cyclization, metal-catalysed reactions and various electrochemical procedures.^6^

**Fig. 1:**
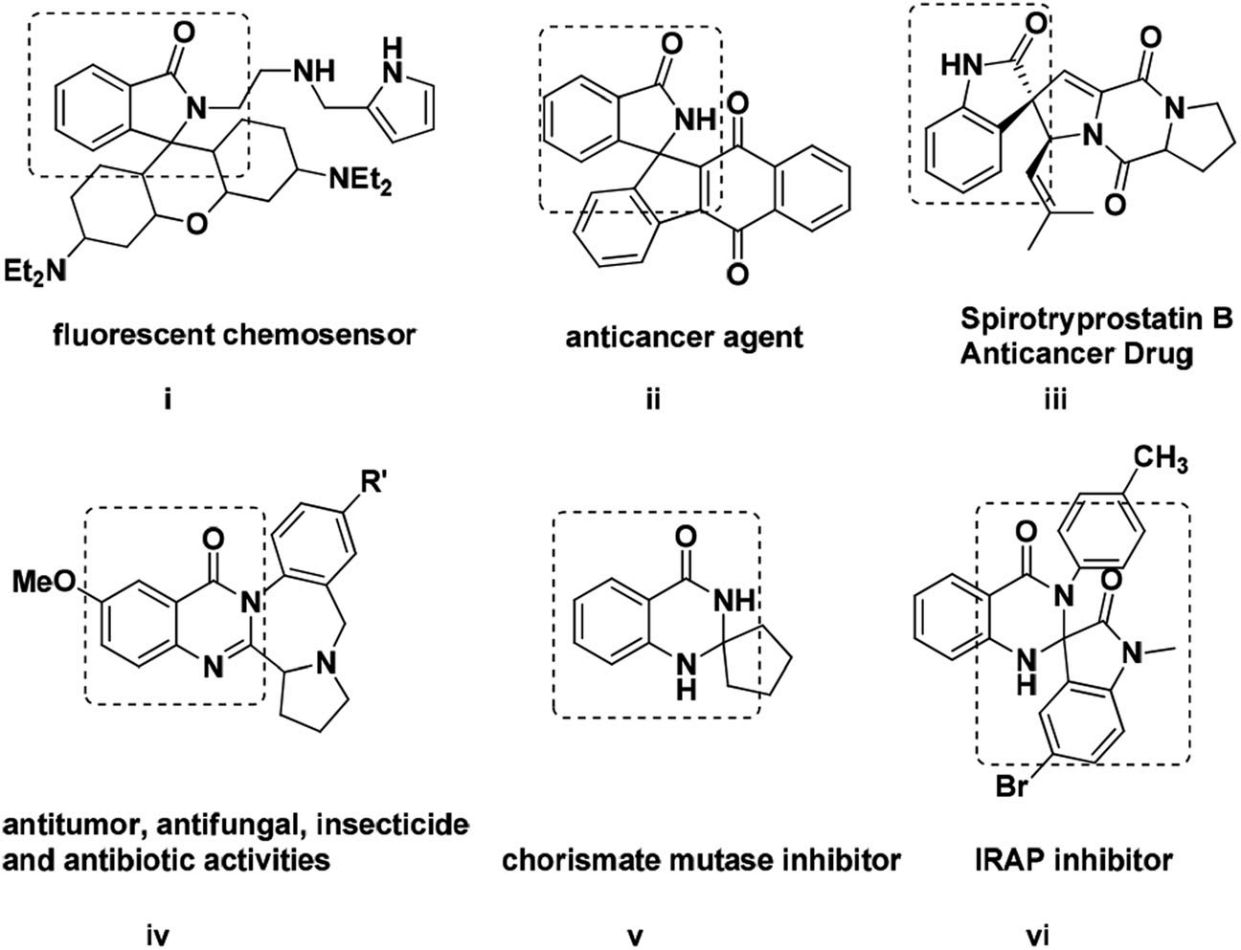
Some biologically active spiro-isoindolinone and quinazolinone units.

Previously, a number of metal catalyzed reactions have also been reported for the spiroannulations.^7^ Among all, Nishimura *et al.* developed an Ir(i) catalyzed [3 + 2] annulation of benzosultam and *N*-acylketimines with 1,3-dienes *via* C–H activation for the synthesis of aminocyclopentene derivatives. Further, Xingwei Li *et al.* developed a Rh(iii)-catalysed [3 + 2] annulation of cyclic *N*-sulfonyl or *N*-acyl ketimines with activated alkenes for the preparation of various spirocyclic compounds.^8^ Recently Yangmin Ma *et al.* developed a one pot nano cerium oxide catalyzed synthesis of spiro-oxindole dihydroquinazolinone derivatives (Scheme 1).^5*c*^ However, development of these type of novel compounds is always challenging and more attractive. Indeed, to the best of our knowledge there are no reports for the synthesis of spiro-isoindolinone dihydroquinazolinones. This led us to give more attention to study these compounds.

**Scheme 1:**
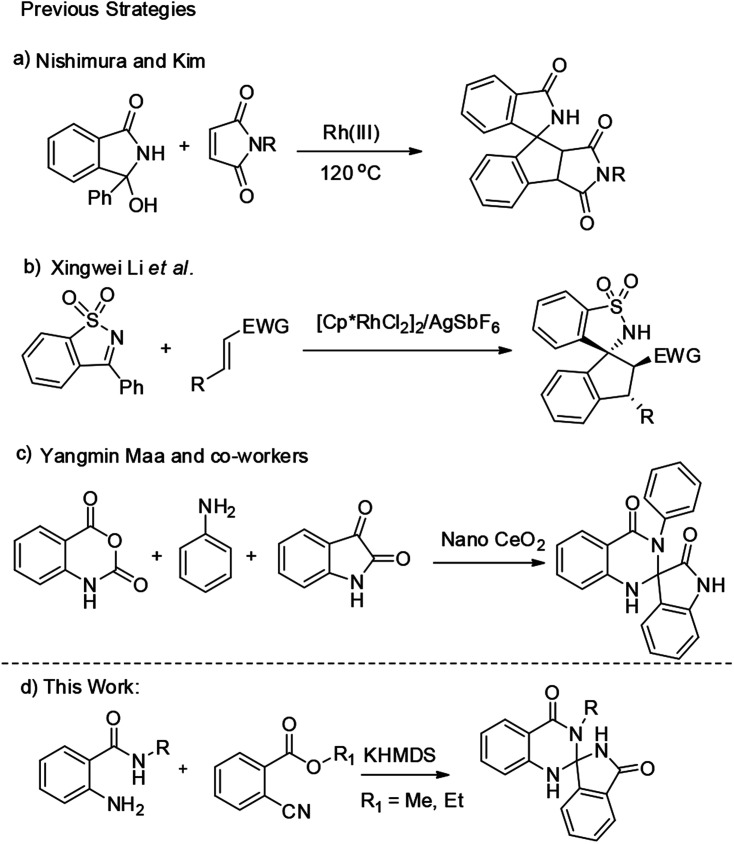
Different strategies for the synthesis of quinazolinone units.

In continuation of our earlier efforts^9^ for the synthesis of various dihydroquinazolinones, herein we would like to report KHMDS mediated synthesis of novel spiro-isoindolinone dihydroquinazolinones. We envisioned the retro synthetic pathway for these compounds, as depicted in Scheme 2. Accordingly these compounds could be synthesised from 2-aminobenzamide and methyl-2-cyanobenzoate or ethyl-2-cyanobenzoate.

**Scheme 2:**
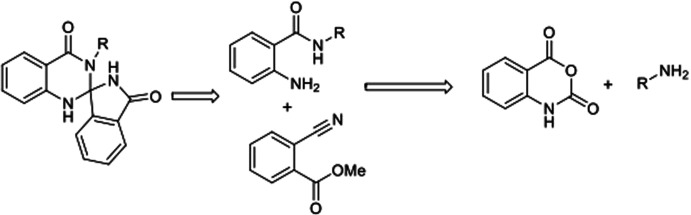
Retro synthetic approach for the synthesis of quinazolinone unit.

Indeed, in order to understand the reaction conditions, we have commenced the reaction by taking 2-amino-*N*-hexyl-benzamide (2) and methyl-2-cyanobenzoate (3) as a model substrates. However, in the initial phase of reaction optimisation, we have screened the reaction in different bases (Fig. 2) and to our delight amongst all the bases KHMDS, LiHMDS and NaHMDS were amenable to get moderate yields. However the reaction had not progressed at low temperatures (5 °C) and could improve the yield at room temperature. Moreover the reaction underwent complete conversion with 1.5 equivalents of base. Further, the reaction was also executed with ethyl-2-cyanobenzoate and could replicate the same yield. Incontinuation, the reaction was futile when the reaction was carried out in DIPEA, DBU, K_2_CO_3_ and Cs_2_CO_3_. Subsequently, the reaction in NaOMe and ^*t*^BuOK produced exclusively the hydrolysis product (4) of methyl-2-cyanobenzoate (Table 1). To circumvent this problem the reaction has also been screened in various solvents.

**Fig. 2:**
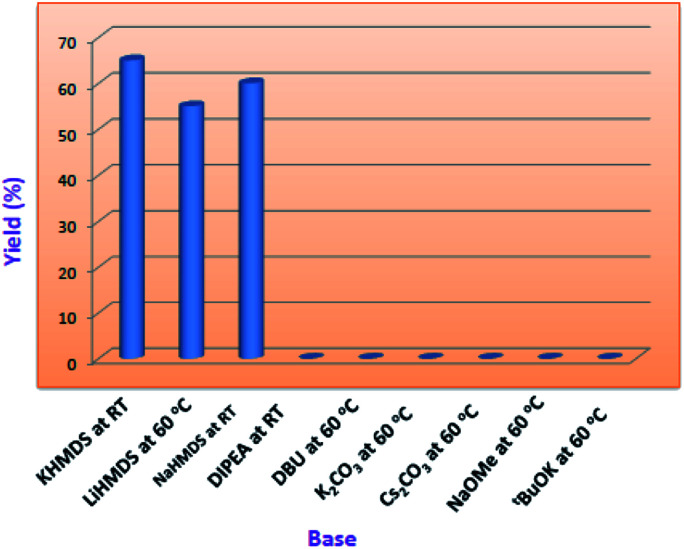
Base screening in 1,4-dioxane.

**Table 1:** Optimisation of the base-mediated spiroannulation^a^

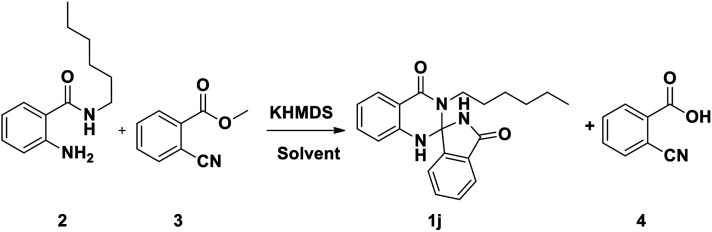				
Entry	Base	Solvent	1j^b^ (%)	Cyano benzoic acid (4)(%)
1	KHMDS	1,4-Dioxane	60	4
2	NaHMDS	1,4-Dioxane	58	3
3	NaOMe	1,4-Dioxane	—	60
4	^*t*^BuOK	1,4-Dioxane	—	60
5	KHMDS	THF	40	5
6	KHMDS	1,2-DME	60	5
7	LiHMDS	1,4-Dioxane	55	5

a Reaction conditions: KHMDS (1 M, 1.5 mmol), 2-amino-benzamide (1 mmol) and methyl-2-cyanobenzoate (1.5 mmol) in 1,4-dioxane (10 mL).

b Isolated yield.

Gratifyingly, among all the solvents 1,4-dioxane, THF and 1,2-dimethoxyethane were found to get good to moderate yields. Whereas, other solvents like DCM ended up with non-polar spots where as in toluene unknown polar impurity was observed. However, there is no reaction progress observed in the presence of trifluoroacetic acid as well as in BF_3_·Et_2_O as a solvent (Fig. 3).

**Fig. 3:**
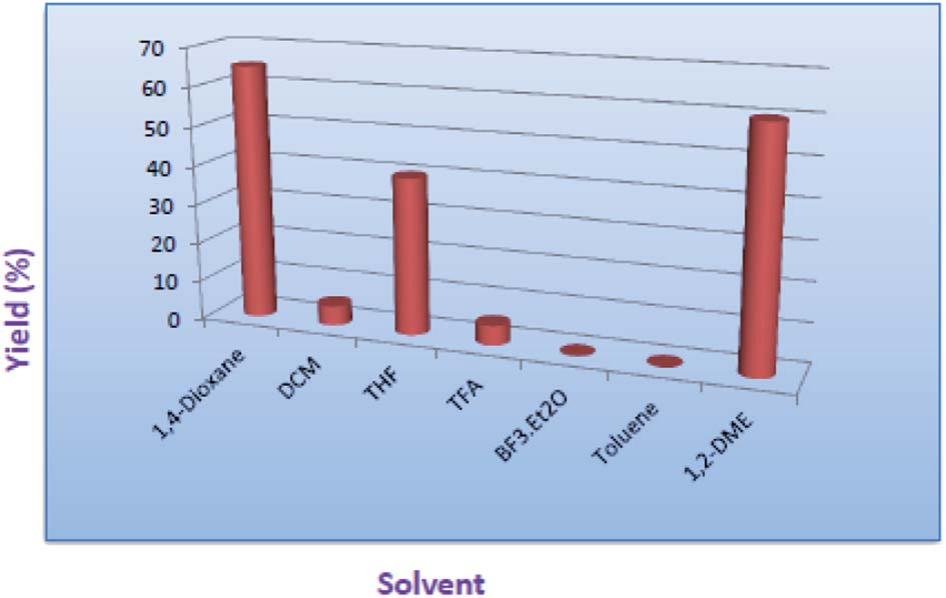
Solvent screening in the presence of KHMDS.

With the optimized conditions in hand, we have explored the applicability of our reaction with various substrates by taking various groups like alkyl, cyclopropyl, cyclohexyl, cycloheptyl, benzyl, naphthyl, furan and to our delight all the substrates were well tolerated under the aforementioned optimal conditions (Table 2). The only limitation of this reaction is substituted cyanobenzoate.

**Table 2:** Substrate scope of the reaction^a^

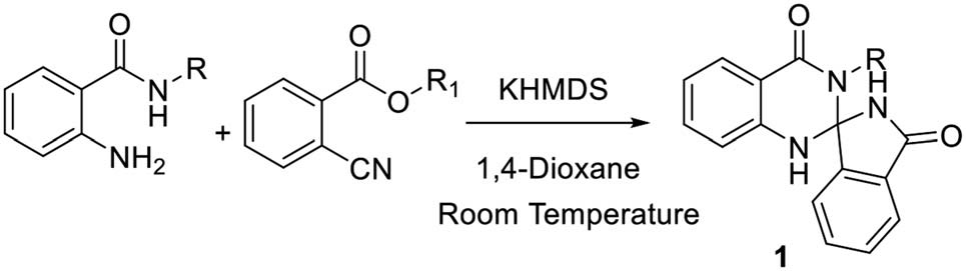
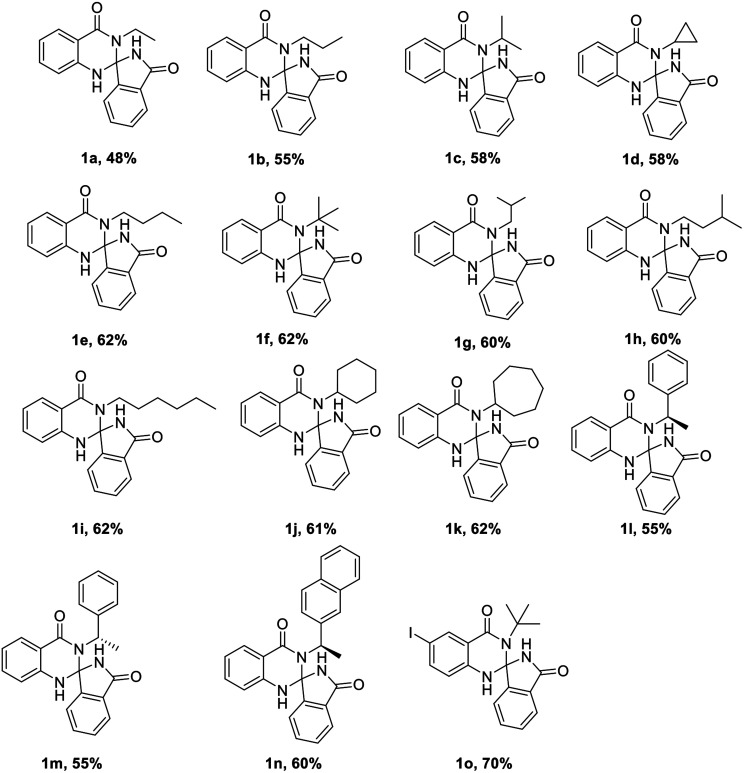

a Reaction conditions: KHMDS (1 M, 1.5 mmol), 2-amino-benzamide (1 mmol) and methyl/ethyl-2-cyanobenzoate (1.5 mmol) in 1,4-dioxane (10 mL).

Based on the aforementioned studies and the literature reports, a plausible mechanism for this reaction has been predicted (Scheme 3). Indeed, to gain insight into the mechanism a series of control experiments have been executed under the similar reaction conditions. Initially the reaction has been carried out without base and both the starting materials were intact. Further the reaction without 2-aminobenzamide resulted hydrolysis product. To explore further, the reaction has also been executed on a 10 gram-scale for the synthesis of 1j and has successfully been demonstrated under the aforementioned optimized conditions.

**Scheme 3:**
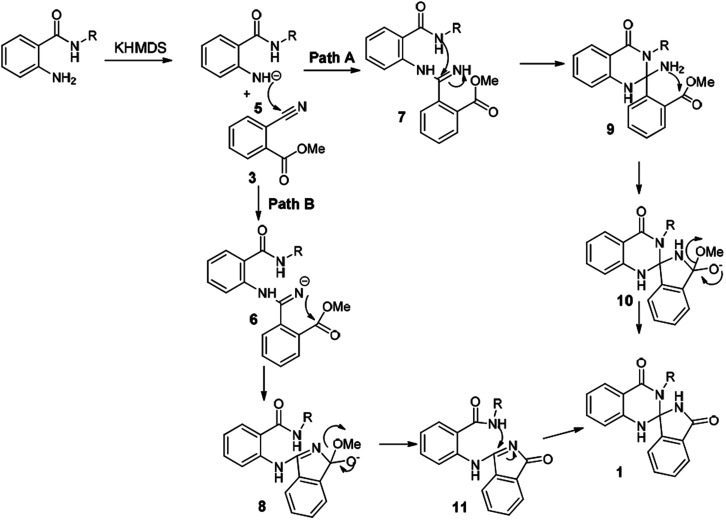
Plausible mechanisms for the synthesis of spiro-isoindolinone dihydroquinazolinones.

The Scheme 3 describes a plausible mechanism for the preparation of compound 1. Initially, KHMDS will abstract N–H proton of amide and nucleophilic nitrogen will attack the cyanobenzoate to get imine intermediate 6 and 7, which on subsequent cyclization lead to the formation of 8 and 9. Finally, the compounds 8 and 9 underwent cyclization to get the spiro-isoindolinone dihydroquinazolinone 1.

## Conclusion

In summary we have successfully synthesized atom economic, scalable and novel spiro-isoindolinone dihydroquinazolinones under mild conditions. The substrate scope of the reaction has been studied to get moderate yields and a possible mechanism for this reaction is proposed. Further investigation of these types of model substrates is underway in our laboratory.

## Conflicts of interest

There are no conflicts to declare.

## Supplementary Material

RA-010-C9RA09567E-s001
