# Supplementary material for: Base mediated spirocyclization of quinazoline: one-step synthesis of spiro-isoindolinone dihydroquinazolinones
Source: RSC Adv. 2020 Mar 4;10(16):9486–91. doi: 10.1039/c9ra09567e (PMC9050170; doi:10.1039/c9ra09567e)
Supplement: RA-010-C9RA09567E-s001 [file RA-010-C9RA09567E-s001.pdf]

Electronic Supplementary Material (ESI) for RSC Advances.
This journal is © The Royal Society of Chemistry 2020

Supporting Information

Base mediated spirocyclization of quinazoline: one-step synthesis of spiro-isoindolinone dihydroquinazolinones

Rapolu Venkateshwarlu,[a,b] V. Narayana Murthy, [a] Krishnaji Tadiparthi,[c] Satish P. Nikumbh,[a] Rajesh Jinkala,[a] Vidavalur Siddaiah,[b] M. V. Madhu babu,[a] Hindupur Ramamohan,[a] Akula. Raghunadh[a*]

[a]*Technology Development Centre, Custom Pharmaceutical Services, Dr. Reddy's Laboratories Ltd, Hyderabad 500049, India*

[b]*Department of Organic Chemistry and FDW, Andhra University, Visakhapatnam 530045, India.*

[c]*Department of Chemistry, Christ deemed to be University, Hosur road, Bangalore 560029, India*

E-mail: raghunadha@drreddys.com

General information……………………………………….……….……….……………S2

Experimental Procedures and Characterizationdata……….………………………………S2

^{1}H and ^{13}C NMR Spectra…………………………………..………..………………...S3

General information

Unless stated otherwise, solvents and chemicals were obtained from commercial sources and used without further purification. Reactions were monitored by thin layer chromatography (TLC) on silica gel plates (60 F254) using EtOAc-Hexane as eluent and visualizing with ultraviolet light or iodine spray. Flash chromatography was performed on silica gel (230-400 mesh) using hexane and ethyl acetate. ^{1}H and ^{13}C NMR spectra were recorded in DMSO-d_6 solution by using a 400 MHz spectrometer. Proton chemical shifts (δ) are relative to tetramethylsilane (TMS, δ = 0.00) as internal standard and expressed in ppm. Spin multiplicities are given as s (singlet), d (doublet), t (triplet) and m (multiplet) as well as b (broad). Coupling constants (*J*) are given in hertz. Infrared spectra were recorded on a FT-IR spectrometer. Melting points were determined using melting point B-540 apparatus and are uncorrected. HRMS was determined using waters LCT premier XETOF ARE-047 apparatus.

Experimental Procedures and Characterization data

General Procedure:

KHMDS (1M, 1.5 mmol) was added slowly to a solution of 2-aminobenzamide (1 mmol) and methyl-2-cyanobenzoate (1.5 mmol) in 1,4-dioxane (10 mL) under nitrogen atmosphere at 25-35°C and stirred for 4-5h. The reaction was monitored by TLC and after the completion of reaction, the reaction mass was diluted with water (5 mL) and extracted with ethyl acetate (3 x 20 mL). The organic layer was washed with 5% HCl (5 mL) followed by water (2 x 10 mL). The combined organic layer was dried over anhydrous sodium sulphate and the solvent was evaporated to get the crude residue which was purified by column chromatography (EtOAc-Hexane) to get pure compound.

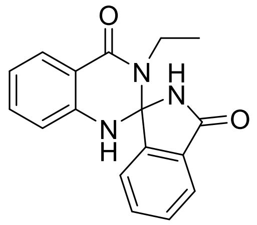

1a

3'-Ethyl-1'*H*-spiro[isoindoline-1,2'-quinazoline]-3,4'(3'*H*)-dione (1a): Light brown color solid; Yield: 48%; ^{1}H NMR (400 MHz, $CDCl_3$): **δ**8.28 (d, J = 8.4 Hz, 1H), 8.22 (bs, 1H), 8.01 (d, J = 7.6 Hz, 1H), 7.94 (d, J = 7.2 Hz, 1H), 7.83-7.74 (m, 3H), 7.51 (d, J = 7.6 Hz, 1H), 7.34 (d, J = 8.4 Hz, 1H), 6.99 (d, J = 8.0 Hz, 1H), 3.47-3.40 (m, 2H), 1.17 (t, J = 7.2 Hz, 3H); ^{13}C NMR (100 MHz, $CDCl_3$); **δ** 167.7, 165.4, 149.8, 144.9, 135.5, 133.9, 133.1, 131.9 (2C), 131.1, 126.7, 125.8, 124.1, 121.9, 120.5, 34.5, 14.7; ESMS-Mass: 294.1 (M+H); HRMS (ESI): Anal. calcd for $C_{17}H_{15}N_3O_2$ $(M+H)^+$ 294.1243, found 294.1232.

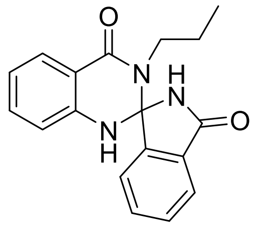

1b

3'-Propyl-1'*H*-spiro[isoindoline-1,2'-quinazoline]-3,4'(3'*H*)-dione (1b): Light brown color solid; Yield: 55%;^{1}H NMR (400 MHz, $CDCl_3$): **δ** 8.57 (bs, 1H), 8.24 (bs, 1H), 8.17 (d, J = 7.6 Hz, 1H), 7.98 (d, J = 7.2 Hz, 1H), 7.91 (d, J = 7.2 Hz, 1H), 7.83-7.72 (m, 2H), 7.45 (t, J = 7.2 Hz, 1H), 7.35 (t, J = 7.2 Hz, 1H), 6.99-6.91 (m, 1H), 3.34 (q, J_1 = 6.8 Hz, J_2 = 6.0 Hz, 2H), 1.55-1.46 (m, 2H), 0.88 (t, J = 7.6 Hz, 3H); ^{13}C NMR (100 MHz, $CDCl_3$);**δ** 168.2, 165.6, 150.2, 145.1, 135.5, 133.8, 132.9, 131.8, 131.6, 131.3, 126.5, 125.6, 123.9, 121.9, 120.8, 41.5, 22.6, 11.6; HRMS (ESI): Anal. calcd for $C_{18}H_{17}N_3O_2$ $(M+H)^+$ 308.1399, found 308.1407.

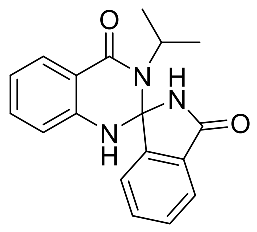

1c

3'-Isopropyl-1'*H*-spiro[isoindoline-1,2'-quinazoline]-3,4'(3'*H*)-dione (1c): Light brown color solid; Yield: 58%; ^{1}H NMR (400 MHz, $CDCl_3$): **δ** 8.24 (d, *J* = 7.6 Hz, 1H), 8.13 (bs, 1H), 8.00 (d, *J* = 7.2 Hz, 2H), 7.93 (d, *J* = 7.2 Hz, 1H), 7.83-7.74 (m, 2H), 7.49 (t, *J* = 7.2 Hz, 1H), 7.32 (t, *J* = 7.6 Hz, 1H), 6.99 (d, *J* = 8.0 Hz, 1H), 4.22-4.13 (m, 1H), 1.16 (d, *J* = 6.4 Hz, 6H); ^{13}C NMR (100 MHz, $CDCl_3$); **δ** 167.8, 164.7, 149.8, 144.9, 135.4, 133.8, 133.0, 131.8, 131.8, 131.1, 126.9, 125.8, 124.1, 121.8, 120.5, 41.4, 22.9 (2C);HRMS (ESI): Anal. calcd for $C_{18}H_{17}N_3O_2$ $(M+H)^+$ 308.1399, found 308.1413.

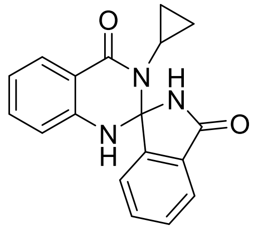

1d

3'-Cyclopropyl-1'*H*-spiro[isoindoline-1,2'-quinazoline]-3,4'(3'*H*)-dione(1d): Off white color solid; Yield: 58%; ^{1}H NMR (400 MHz, DMSO-d_6): **δ** 10.89 (bs, 1H), 8.34 (d, *J*= 3.6 Hz, 1H), 8.02 (d, *J*= 7.2 Hz, 1H), 7.86-7.74 (m, 3H), 7.65-7.62 (m, 1H), 7.48-7.44 (m, 1H), 7.27-7.20 (m, 1H), 7.04-7.01 (m, 1H), 2.80-2.70 (m, 1H), 0.66-0.57(m, 2H), 0.42-0.37 (m, 2H); ^{13}C NMR (100 MHz, DMSO-d_6): **δ** 169.1, 164.6, 150.6, 145.3, 135.5, 133.7, 132.8, 131.1, 130.9, 129.5, 127.4, 124.4, 123.1, 122.1,121.7, 50.1, 28.5 (2C);HRMS (ESI): Anal. calcd for $C_{18}H_{15}N_3O_2$ $(M+H)^+$ 306.1243, found 306.1253.

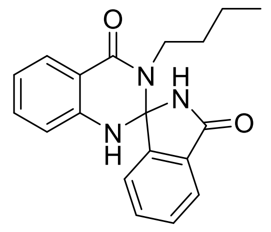

1e

3'-Butyl-1'*H*-spiro[isoindoline-1,2'-quinazoline]-3,4'(3'*H*)-dione(1e): Off white color solid; Yield: 62%; ^{1}H NMR (400 MHz, $CDCl_3$): **δ** 8.22 (d, *J*= 7.2 Hz, 1H), 8.00 (d, *J*= 7.2 Hz, 1H), 7.93 (d, *J*= 7.2 Hz, 1H), 7.82-7.73 (m, 2H), 7.29-7.25 (m, 1H), 6.99 (d, *J*= 7.6 Hz, 1H), 3.40 (t, *J*= 6.4 Hz, 2H), 1.51-0.1.43 (m, 2H), 1.34-1.24 (m, 2H), 0.82 (t, *J*= 7.2 Hz, 3H): ^{13}C NMR (100 MHz, $CDCl_3$): **δ** 167.9, 165.6, 150.1, 145.1, 135.5, 133.8, 133.0, 131.9, 131.8, 131.2, 126.6, 125.7, 123.9, 122.0, 120.7, 39.5, 31.5, 20.2, 13.6;HRMS (ESI): Anal. calcd for $C_{18}H_{15}N_3O_2$ $(M+H)^+$ 322.1556, found 322.1550.

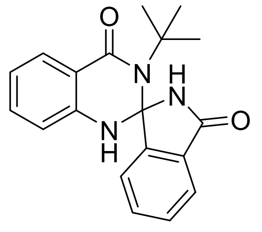

1f

3'-(*tert*-Butyl)-1'*H*-spiro[isoindoline-1,2'-quinazoline]-3,4'(3'*H*)-dione (1f): Off white color solid; Yield: 62%; ^{1}H NMR (400 MHz, DMSO-d_6): **δ** 8.13 (s, 1H), 8.04 (d, *J* = 3.2 Hz, 1H), 7.88-7.85 (m, 3H), 7.82-7.78 (m, 1H), 7.48 (t, *J* = 7.6 Hz, 1H), 7.28-7.22 (m, 1H), 7.21-7.03 (m, 1H), 1.25 (s, 9H); ^{13}C NMR (100 MHz, $CDCl_3$); **δ** 167.8, 164.6, 149.8, 144.8, 135.3, 133.8, 133.0, 131.7, 131.6, 131.1, 127.6, 125.8,124.0, 122.1, 120.6, 51.0, 28.9(3C);ESMS-Mass:322.1(M+H); HRMS (ESI): Anal. calcd for $C_{19}H_{19}N_3O_2$ $(M+H)^+$ 322.1556, found 322.1544.

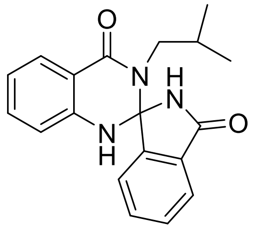

1g

3'-Isobutyl-1'*H*-spiro[isoindoline-1,2'-quinazoline]-3,4'(3'*H*)-dione(1g): Off white color solid; Yield: 60%; ^{1}H NMR (400 MHz, $CDCl_3$): **δ** 8.26-8.21 (m, 2H), 8.19 (bs, 1H), 8.00 (d, *J* = 7.2 Hz 1H), 7.92 (d, *J* = 6.8 Hz, 1H), 7.86-7.77 (m, 2H), 7.33-7.26 (m, 1H), 7.49-7.46 (m,1H), 6.99 (d, *J* = 7.6 Hz, 1H), 3.24-3.21 (t, *J* = 6.0 Hz, 2H), 1.80-1.74 (m,1H), 0.89-0.88 (d, *J* = 4.4 Hz, 6H); ^{13}C NMR (100 MHz, $CDCl_3$); **δ** 167.8, 165.6, 150.2, 145.1, 135.4, 133.7, 133.0, 131.9, 131.4, 125.9, 125.8, 123.9, 122.1, 120.7, 47.3, 28.4, 20.3; ESMS-Mass:350.18 (M+H); HRMS (ESI): Anal. calcd for $C_{19}H_{24}NO_4$ $(M+H)^+$ 322.1556, found 322.1544.

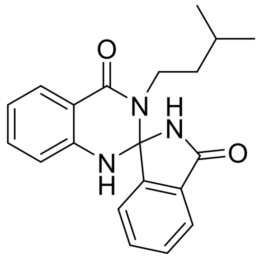

1h

3'-Isopentyl-1'*H*-spiro[isoindoline-1,2'-quinazoline]-3,4'(3'*H*)-dione (1h): Off white color solid; Yield: 60%; ^{1}H NMR (400 MHz, $CDCl_3$): **δ** 8.25 (d, *J* = 8.0 Hz, 1H), 8.13 (bs, 1H), 8.03-799 (m, 2H), 7.93 (d, *J* = 7.2 Hz, 1H), 7.80-7.77 (m, 2H), 7.49-7.45 (m, 1H), 7.31 (t, *J* = 7.2 Hz, 1H), 6.98 (d, *J* = 7.6 Hz, 1H), 3.43-3.38 (m, 2H), 1.59-1.53 (m, 1H), 1.40-1.35 (m, 2H), 0.82 (d, *J* = 6.4 Hz, 6H); ^{13}C NMR (100 MHz, $CDCl_3$): **δ** 167.8, 165.5, 150.1, 145.1, 135.4, 133.8, 133.1, 131.9, 131.9, 131.1, 126.6, 125.8, 124.0, 122.1, 120.6, 38.3, 37.9, 25.7, 22.3 (2C); ESMS-Mass:350.18 (M+H); HRMS (ESI): Anal. calcd for $C_{19}H_{24}NO_4$ $(M+H)^+$ 336.1712, found 336.1697.

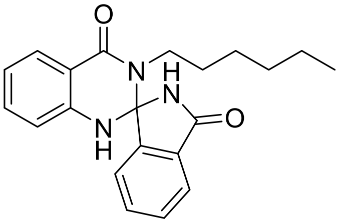

1i

3'-Hexyl-1'*H*-spiro[isoindoline-1,2'-quinazoline]-3,4'(3'*H*)-dione (1i): Light brown color solid; Yield: 62% ; ^{1}H NMR (400 MHz, $CDCl_3$): **δ** 8.30 (bs, 1H), 8.21-8.17 (m, 2H), 8.00 (d, *J*= 6.8 Hz, 1H), 7.93 (d, *J* = 7.2 Hz, 1H), 7.81-7.73 (m, 2H), 7.47 (t, *J* = 7.2 Hz, 1H), 7.28 (t, *J* = 7.2 Hz, 1H), 6.98 (d, *J* = 7.6 Hz, 1H), 3.39-3.34 (m, 2H), 1.51-1.43 (m, 2H), 1.24-1.18 (m, 2H),1.14-1.12 (m, 4H), 0.81 (t, *J* = 6.8 Hz, 3H); ^{13}C NMR (100 MHz, $CDCl_3$): **δ** 167.9, 165.5, 150.1, 145.0, 135.5, 133.8, 133.0, 131.9, 131.8, 131.1, 126.5, 125.7, 123.9, 121.9, 120.7,39.8,31.5, 29.4, 26.8, 22.4, 13.9;HRMS (ESI): Anal. Calc.Mass for $C_{21}H_{24}N_3O_2$ $(M+H)^+$ 350.1869, found 350.1885.

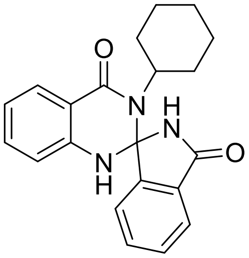

1j

3'-Cyclohexyl-1'*H*-spiro[isoindoline-1,2'-quinazoline]-3,4'(3'*H*)-dione (1j): Light brown color solid; Yield: 61%; ^{1}H NMR (400 MHz, $CDCl_3$): **δ** 8.20 (d, *J* = 8.0 Hz, 1H), 8.13 (d, *J* = 7.6 Hz, 1H), 8.00 (d, *J* =7.2 Hz, 1H), 7.92 (d, *J* = 7.6 Hz, 1H), 7.82-7.73 (m, 2H), 7.46 (t, *J* = 7.6 Hz, 1H),7.28 (t, *J* = 8.0 Hz, 1H), 6.97 (d, *J* = 8.0 Hz, 1H), 3.91-3.87 (m, 1H), 1.91-1.87 (m, 2H), 1.65-1.55 (m, 3H), 1.40-1.37 (m, 2H),1.18-1.09 (m, 3H); 13C NMR (100 MHz, CDCl3); **δ** 167.9, 164.7, 149.9, 144.9, 135.4, 133.8, 133.0, 131.8, 131.8, 131.2, 126.8, 125.7, 123.9, 121.9, 120.6, 48.2, 32.9 (2C), 25.6, 24.6 (2C); HRMS (ESI): Anal. Calc.Mass $C_{21}H_{22}N_3O_2$ $(M+H)^+$ 348.1712, found 348.1721.

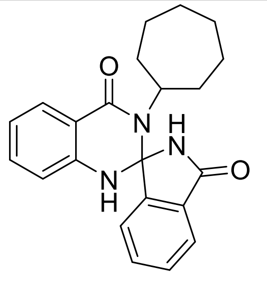

1k

3'-Cycloheptyl-1'*H*-spiro[isoindoline-1,2'-quinazoline]-3,4'(3'*H*)-dione (1k): Light brown color solid; Yield: 62%; ^{1}H NMR (400 MHz, $CDCl_3$): **δ** 8.12-8.05 (m, 2H), 8.02 (bs, 1H), 7.93 (d, *J*= 7.2 Hz, 1H), 7.86 (d, *J* =7.6 Hz, 1H), 7.82-7.74 (m, 2H), 7.47 (t, *J* = 6.8 Hz, 1H), 7.33-7.26 (m, 1H), 6.98 (d, *J* = 8.0 Hz, 1H), 4.10-4.07 (m, 1H), 1.93-1.89 (m, 2H), 1.68-1.50 (m, 4H), 1.45-1.25 (m, 6H); ^{13}C NMR (100 MHz, $CDCl_3$); **δ** 167.8, 164.3, 149.9, 144.9, 135.4, 133.7, 133.0, 131.8, 131.8, 131.2, 126.8, 125.7, 124.0, 122.1, 120.6, 50.4, 35.0 (2C), 28.2 (2C), 23.9 (2C); ESMS-Mass:362.18 (M+H); HRMS (ESI): Anal. calcd for $C_{22}H_{23}N_3O_2$ $(M+H)^+$ 362.1869, found 362.1874.

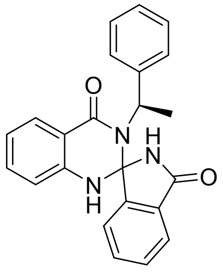

1l

3'-(*R*)-1-Phenylethyl-1'*H*-spiro[isoindoline-1,2'-quinazoline]-3,4'(3'*H*)-dione (1l): Light brown color solid; Yield: 55%; ^{1}H NMR (400 MHz, $CDCl_3$): **δ** 8.64 (bs, 1H), 8.28 (d, *J* = 7.6 Hz, 1H), 7.90 (d, *J* = 7.2 Hz, 1H), 7.84-7.80 (m, 1H), 7.75-7.72 (m, 1H), 7.71-7.61 (m, 2H), 7.50 (t, *J*= 7.6 Hz, 1H), 7.36-7.26 (m, 3H), 7.21-7.16 (m, 3H), 6.98 (d, *J* = 8.0 Hz, 1H), 5.27-5.19 (m, 1H), 1.53 (d, *J* = 6.8 Hz, 3H); ^{13}C NMR (100 MHz, $CDCl_3$); **δ** 167.7, 164.7, 143.4, 135.2, 133.9, 132.9, 132.1 (2C), 130.9, 128.6 (3C), 127.2, 126.7, 126.3 (3C), 125.9, 123.9, 122.2, 120.6, 49.3,

21.8; ESMS-Mass:370.15 (M+H); HRMS (ESI): Anal. calcd for $C_{23}H_{19}N_3O_2$ $(M+H)^+$ 370.1556, found 370.1567.

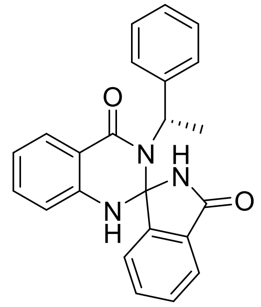

1m

3'-(*S*)-1-Phenylethyl-1'H-spiro[isoindoline-1,2'-quinazoline]-3,4'(3'H)-dione (1m): Light brown color solid; Yield: 55%; ^{1}H NMR (400 MHz, $CDCl_3$): **δ** 8.64 (bs, 1H), 8.25 (d, *J* = 8.0 Hz, 1H), 8.05 (bs, 1H), 7.89 (d, *J* = 7.2 Hz, 1H), 7.73 (t, *J* = 6.8 Hz, 1H), 7.68 (t, *J* = 7.6 Hz, 1H), 7.61 (d, *J* = 7.2 Hz, 1H), 7.49 (t, *J* = 7.2 Hz, 1H), 7.36-7.26 (m, 3H), 7.22-7.17 (m, 3H), 6.98 (d, *J*= 8.0 Hz, 1H), 5.26-5.19 (m, 1H), 1.52 (d, *J* = 6.8 Hz, 3H); ^{13}C NMR (100 MHz, $CDCl_3$); **δ** 167.8, 164.7, 143.3, 135.2, 133.8, 132.8, 132.1 (2C), 130.9, 128.6 (3C), 127.2, 126.6, 126.3 (3C), 125.8, 123.8, 122.1, 120.6, 49.3, 21.8; ESMS-Mass:370.15 (M+H); HRMS (ESI): Anal. calcd for $C_{23}H_{19}N_3O_2$ $(M+H)^+$ 370.1556, found 370.1554.

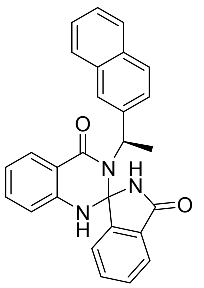

1n

3'-[(*R*)-1-(Naphthalen-2-yl)ethyl]-1'H-spiro[isoindoline-1,2'-quinazoline]-3,4'(3'H)-dione (1n): Light brown color solid; Yield: 60%; ^{1}H NMR (400 MHz, $CDCl_3$): **δ** 8.56 (bs, 1H), 8.33 (d, *J*= 7.6 Hz, 1H), 7.90 (d, *J* = 8.4 Hz, 1H), 7.67 (d, *J* = 7.6 Hz, 1H), 7.60-7.51 (m, 4H), 7.46-7.41 (m, 2H), 7.39-7.35 (m, 3H), 7.33-7.26(m,1H),7.24-7.19 (m, 1H),6.89 (d, *J* = 7.6 Hz, 1H), 6.84 (d, *J* = 7.6 Hz, 1H), 5.98 (t, *J* = 7.2 Hz, 1H), 1.75 (d, *J* = 6.8 Hz, 3H); ^{13}C NMR (100 MHz, $CDCl_3$); **δ** 167.5, 164.6, 149.8, 145.2, 137.9, 134.4, 133.7, 133.6, 132.3, 132.2, 132.0, 131.0, 130.4, 128.5, 128.4, 126.2, 126.1, 125.8, 125.5, 124.8, 123.5, 123.2, 122.7, 121.3, 120.5, 45.2, 19.9; ESMS-Mass:420.17 (M+H); HRMS (ESI): Anal. calcd for $C_{27}H_{21}N_3O_2$ $(M+H)^+$ 420.1712, found 420.1711.

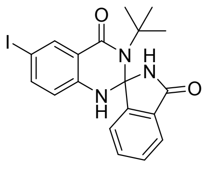

1o

3'-(tert-butyl)-6'-iodo-1'H-spiro[isoindoline-1,2'-quinazoline]-3,4'(3'H)-dione(1o): Light brown white color solid; Yield: 70%; ^{1}H NMR (400 MHz, $CDCl_3$): □ 8.57 (s, 1H), 8.46-8.45 (d, *J* = 8.4 Hz, 1H), 8.15 (s, 1H), 7.97-7.792(m, 1H), 7.80-7.70 7.28-7.22 (m, 1H), 6.75-6.72 (m, 1H), 1.34 (s, 9H); ^{13}C NMR (100 MHz, $CDCl_3$); □ 166.4,162.0,148.9, 143.4,139.5,139.4,134.4,133.1,132.9,132.2,128.1,123.1,121.4,121.1, 88.9, 50.1, 27.8 (3C); ESMS-Mass: 447.28 (M+H); HRMS (ESI): Anal. calcd for $C_{19}H_{19}IN_3O_2$ $(M+H)^+$ 448.0522, found 448.0516.

Spectra Data

^{1}H and ^{13}C NMR Spectra

^{1}H NMR of 3'-Ethyl-1'H-spiro [isoindoline-1, 2'-quinazoline]-3, 4' (3'H)-dione (1a):

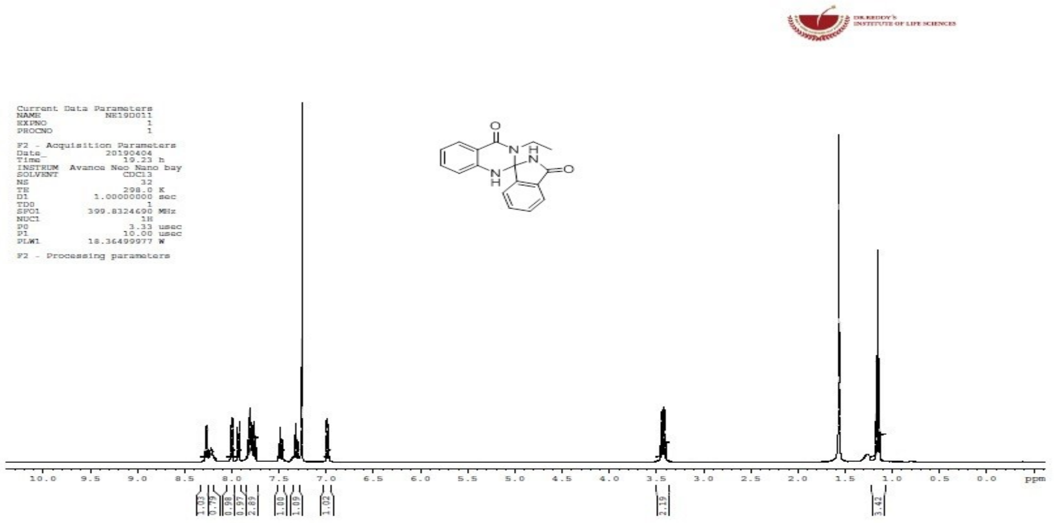

^{13}C NMR of 3'-Ethyl-1'H-spiro [isoindoline-1, 2'-quinazoline]-3, 4' (3'H)-dione (1a):

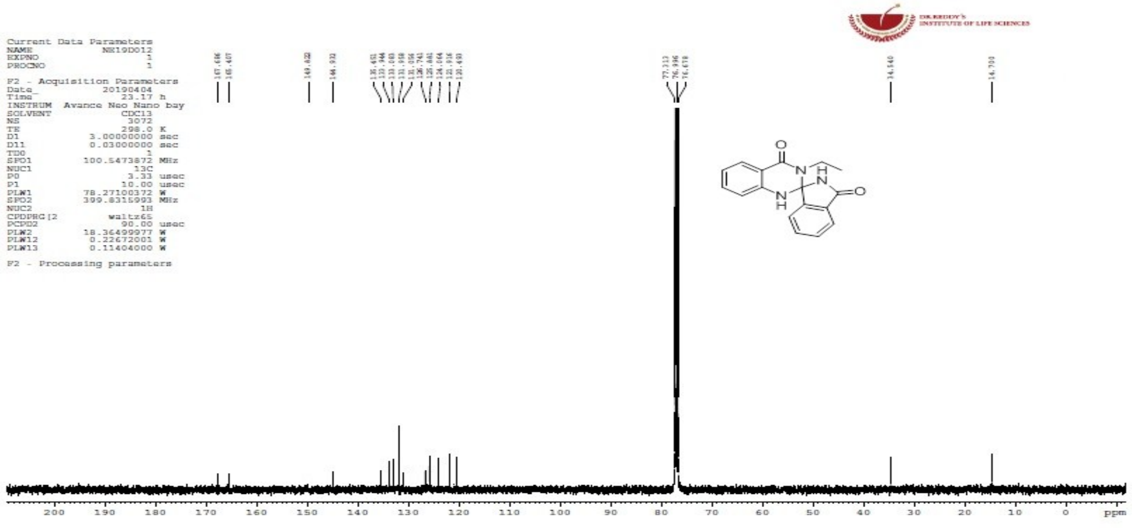

HRMS of 3'-Ethyl-1'H-spiro [isoindoline-1, 2’-quinazoline]-3, 4’ (3'H)-dione (1a):

Elemental Composition Report — Page 1

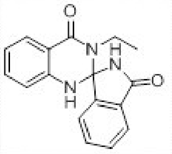

Single Mass Analysis
Tolerance = 5.0 PPM / DBE: min = -1.5, max = 80.0
Element prediction: Off
Number of isotope peaks used for i-FIT = 3

Monoisotopic Mass, Even Electron Ions
149 formula(e) evaluated with 1 results within limits (all results (up to 1000) for each mass)
Elements Used:
C: 0-28 H: 0-37 N: 0-5 O: 0-7
SR/RV-012-0328993
190315001 21 (0.262) Cm (21:23-(33:36+5:6))

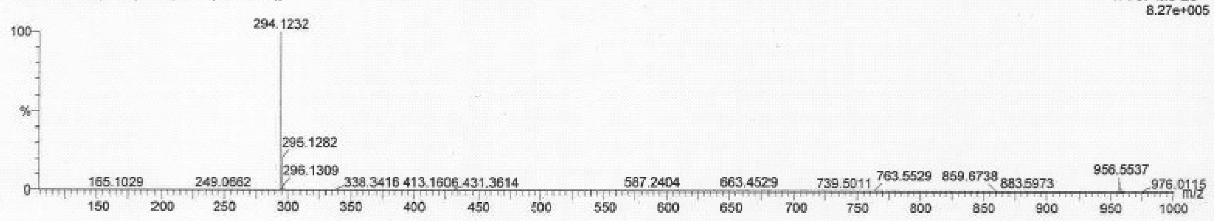

Minimum:				-1.5		
Maximum:		5.0	5.0	80.0		
Mass	Calc. Mass	mDa	PPM	DBE	i-FIT	Formula
294.1232	294.1243	-1.1	-3.7	11.5	292.0	C17 H16 N3 O2

f 3'-Propyl-1'H-spiro [isoindoline-1, 2’-quinazoline]-3, 4’(3'H)-dione (1b):

```
Current Data Parameters
NAME          NE19E176
EXPNO                1
PROCNO               1

F2 - Acquisition Parameters
Date_         20190527
Time           12.58 h
INSTRUM  Avance Neo Nano bay
SOLVENT          CDCl3
NS                  16
TE             301.1 K
D1      1.00000000 sec
TD0                  1
SFO1    399.8324690 MHz
NUC1                1H
P0              3.33 usec
P1             10.00 usec
PLW1     18.36499977 W

F2 - Processing parameters
```

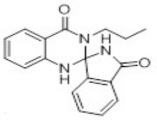

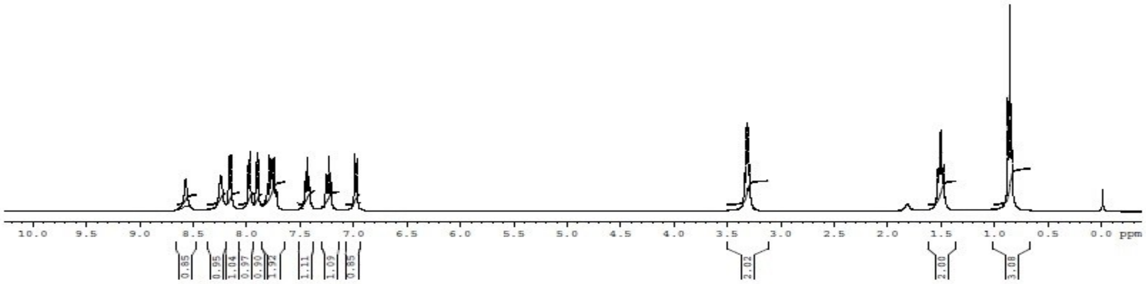

^{13}C NMR of 3'-Propyl-1'H-spiro [isoindoline-1, 2'-quinazoline]-3, 4'(3'H)-dione (1b):

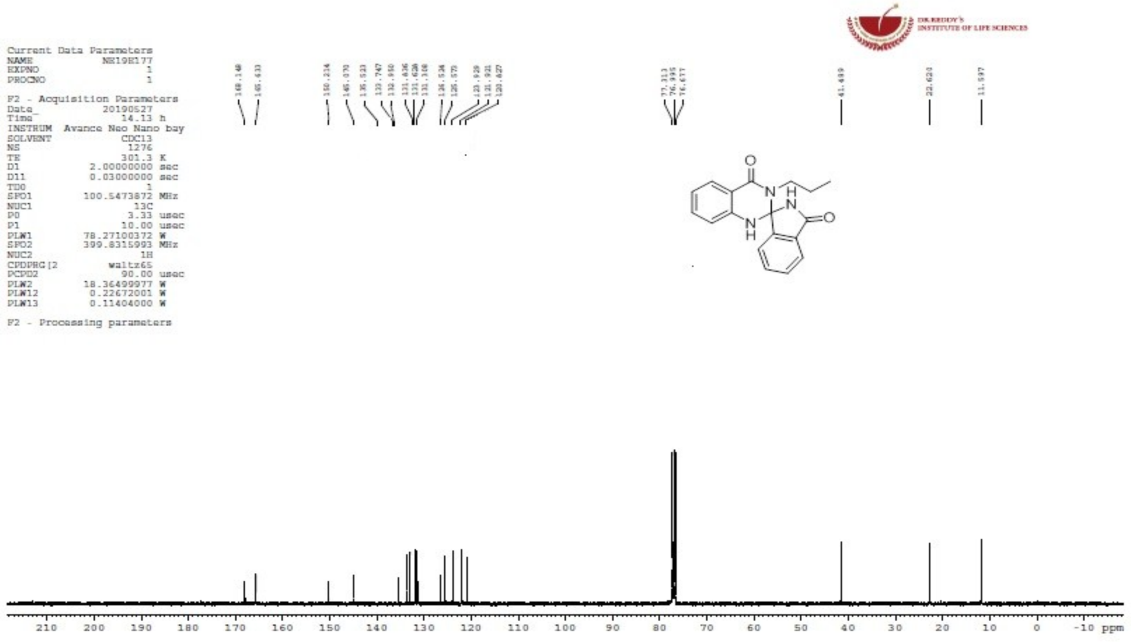

HRMS of 3'-Propyl-1'H-spiro [isoindoline-1, 2'-quinazoline]-3, 4'(3'H)-dione (1b):

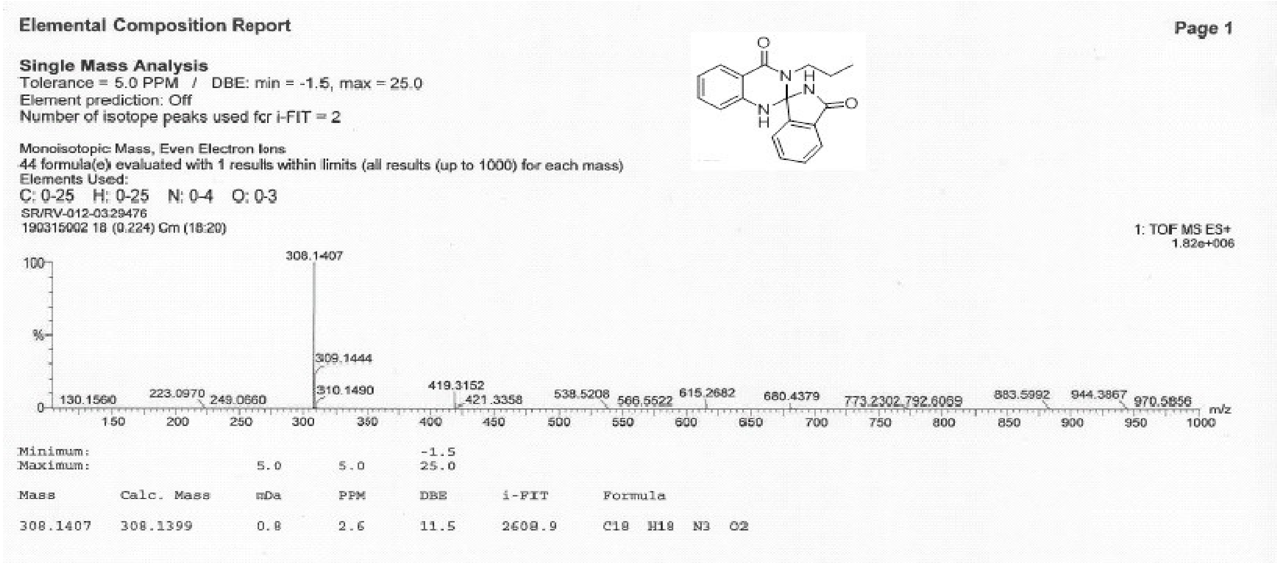

Elemental Composition Report

Page 1

Single Mass Analysis
Tolerance = 5.0 PPM / DBE: min = -1.5, max = 25.0
Element prediction: Off
Number of isotope peaks used for i-FIT = 2

Monoisotopic Mass, Even Electron Ions
44 formula(e) evaluated with 1 results within limits (all results (up to 1000) for each mass)
Elements Used:
C: 0-25 H: 0-25 N: 0-4 O: 0-3
SR/RV-012-0329476
190315002 18 (0.224) Cm (18:20)

1: TOF MS ES+
1.82e+006

Minimum:				-1.5		
Maximum:		5.0	5.0	25.0		
Mass	Calc. Mass	mDa	PPM	DBE	i-FIT	Formula
308.1407	308.1399	0.8	2.6	11.5	2608.9	C18 H18 N3 O2

¹H NMR of 3'-Isopropyl-1'H-spiro[isoindoline-1, 2'-quinazoline]-3,4'(3'H)-dione (1c):

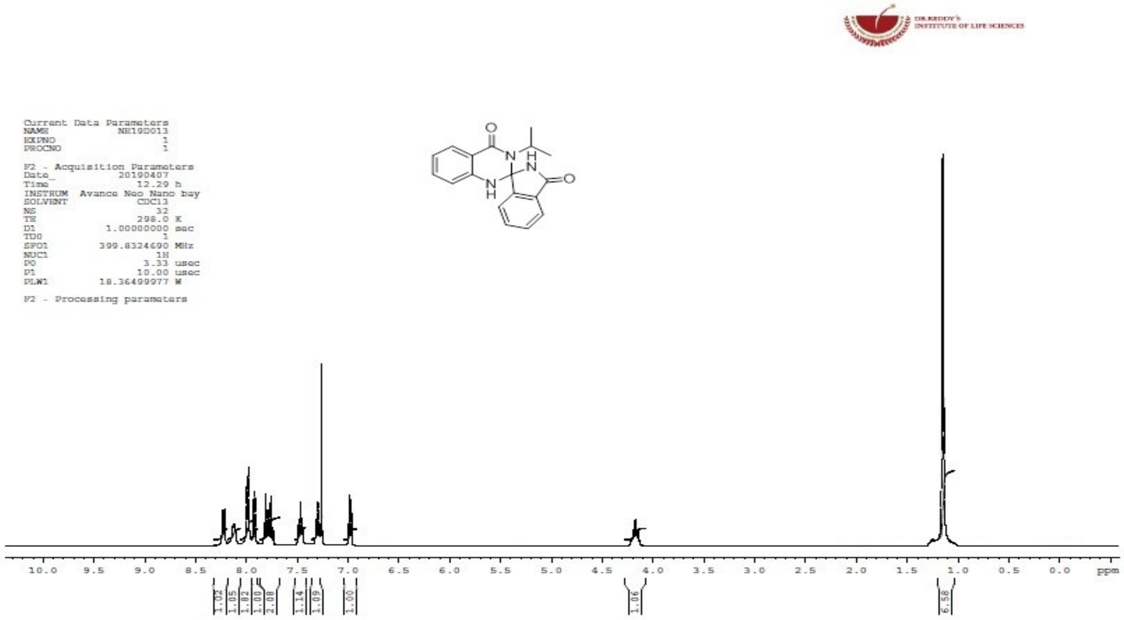

¹³C NMR of 3'-Isopropyl-1'H-spiro[isoindoline-1,2'-quinazoline]-3,4'(3'H)-dione(1c):

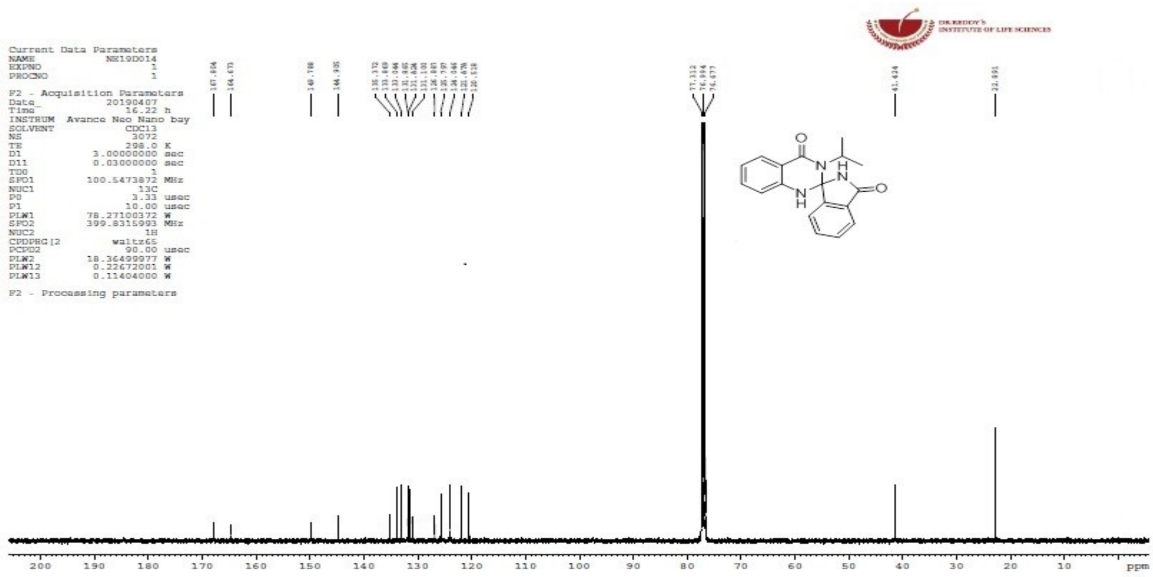

HRMS of 3'-Isopropyl-1'H-spiro[isoindoline-1,2'-quinazoline]-3,4'(3'H)-dione (1c):

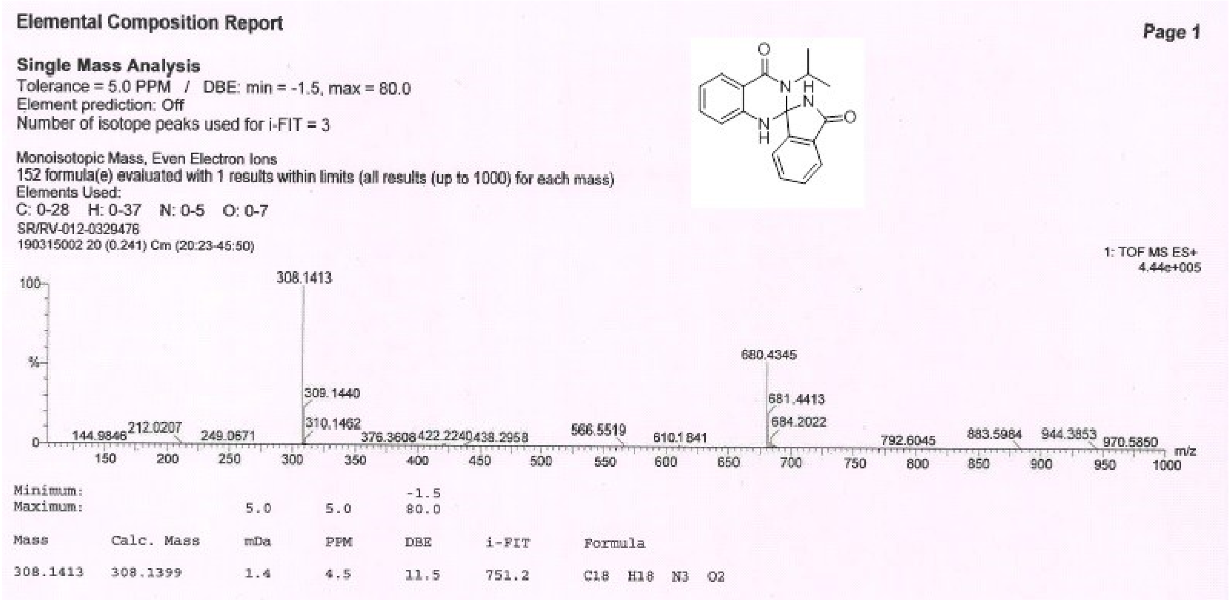

^{1}H NMR of 3'-Cyclopropyl-1'H-spiro[isoindoline-1,2'-quinazoline]-3,4'(3'H)-dione(1d)

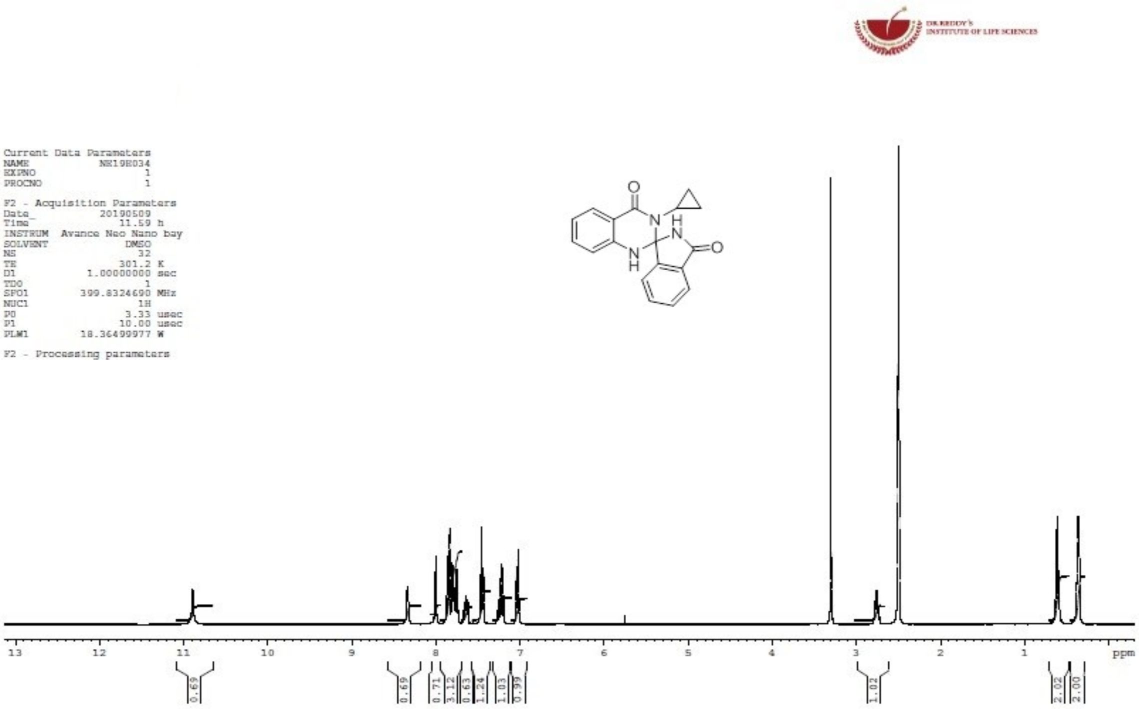

^{13}C NMR of 3'-Cyclopropyl-1'H-spiro [isoindoline-1, 2’-quinazoline]-3, 4’ (3'H)-dione(1d)

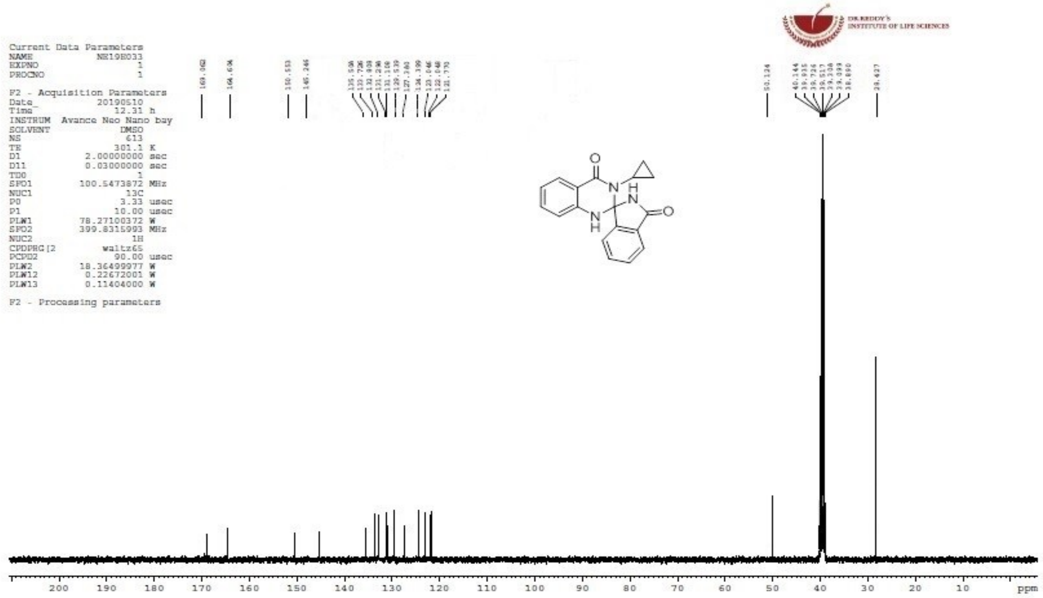

HRMS of 3'-cyclopropyl-1'H-spiro[isoindoline-1,2'-quinazoline]-3,4'(3'H)-dione(1d)

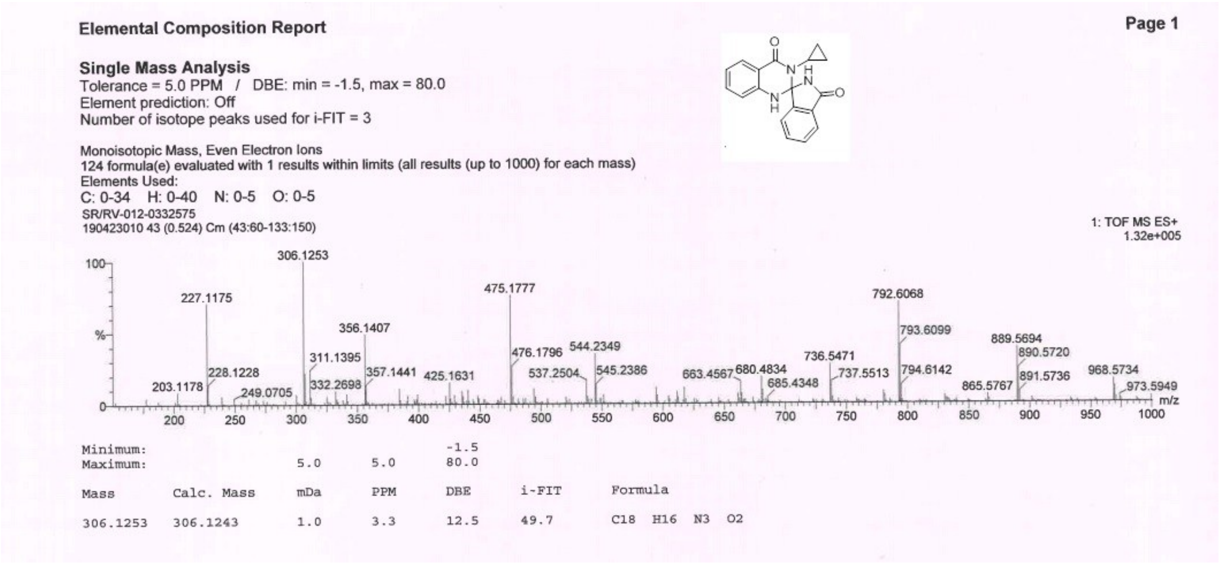

^{1}H NMR of 3'-Butyl-1'H-spiro[isoindoline-1, 2’-quinazoline]-3, 4’ (3'H)-dione(1e):

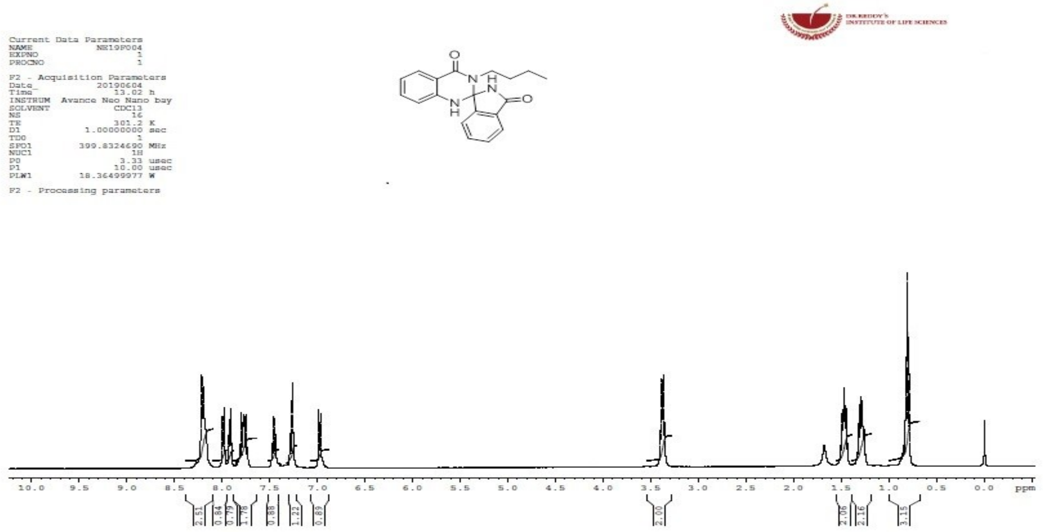

^{13}C NMR of 3'-Butyl-1'H-spiro[isoindoline-1,2'-quinazoline]-3,4'(3'H)-dione(1e):

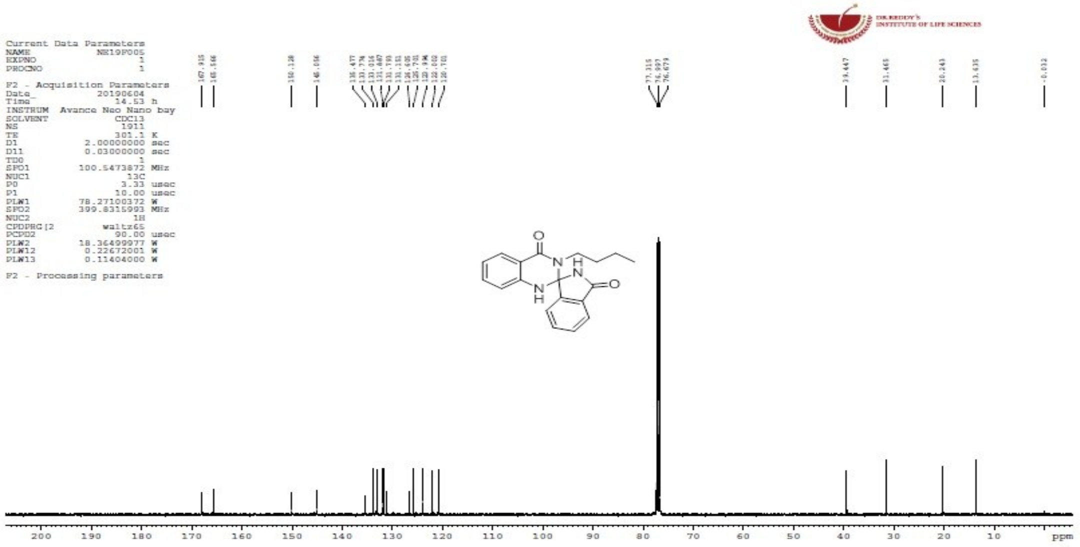

HRMS of 3'-Butyl-1'H-spiro[isoindoline-1,2'-quinazoline]-3,4'(3'H)-dione(1e):

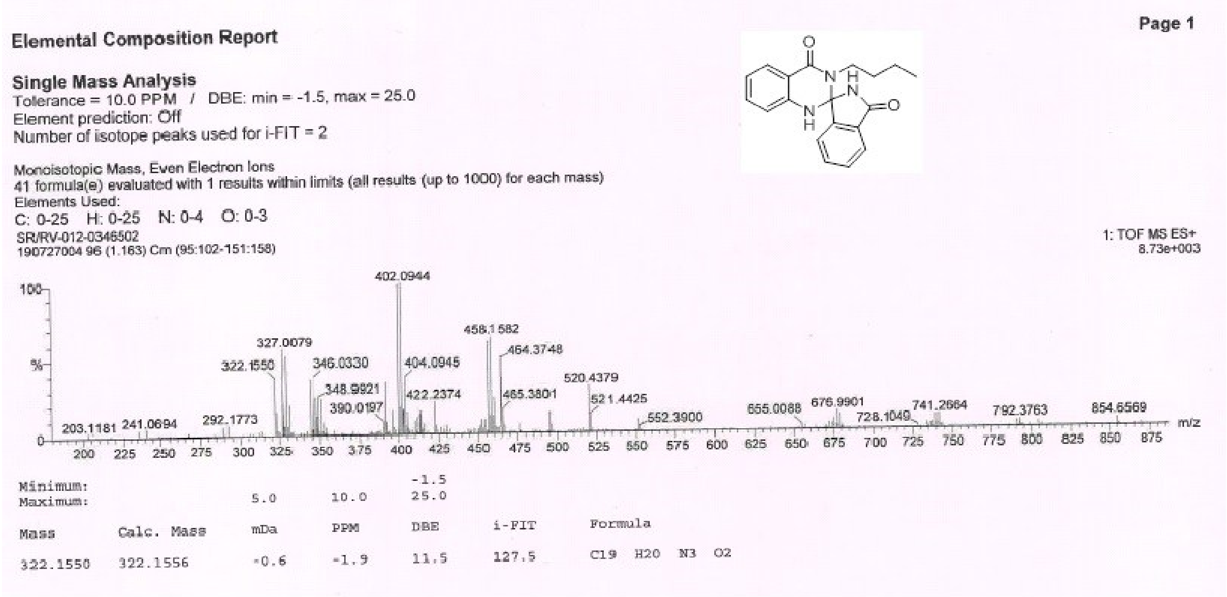

1

H-NMR of 3'-(tert-butyl)-1'H-spiro[isoindoline-1,2'-quinazoline]-3,4'(3'H)-dione (1f):

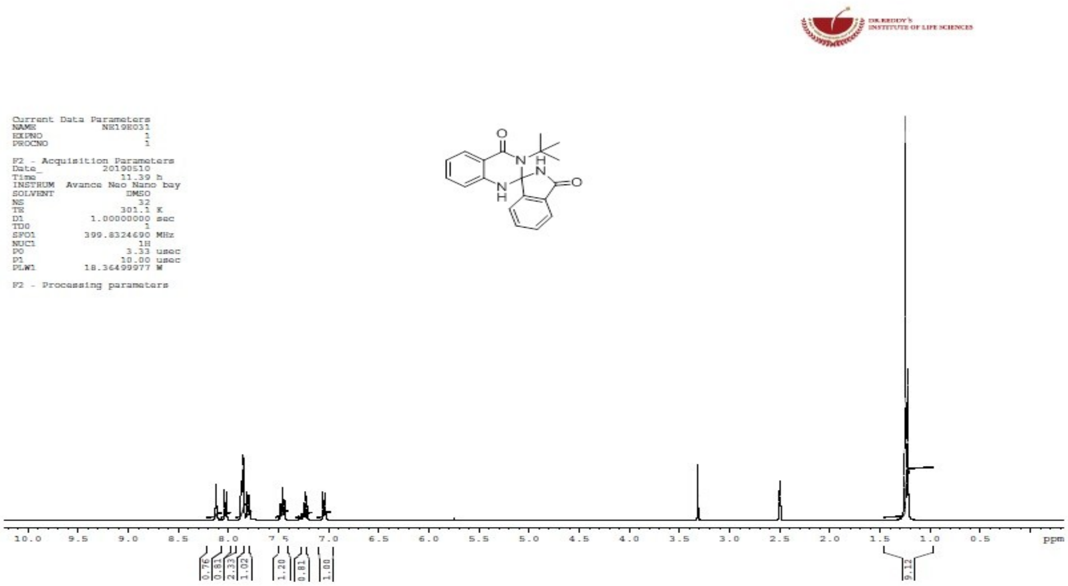

^{13}C-NMR of 3'-(tert-butyl)-1'H-spiro[isoindoline-1,2'-quinazoline]-3,4'(3'H)-dione (1f):

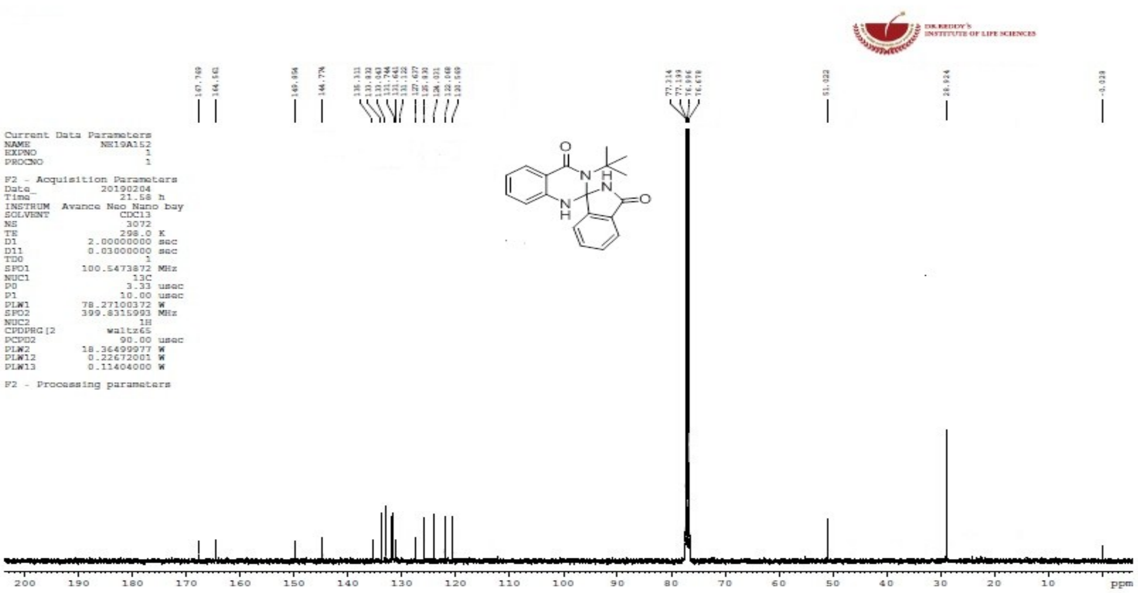

HRMS of 3'-(tert-butyl)-1'H-spiro[isoindoline-1,2'-quinazoline]-3,4'(3'H)-dione (1f):

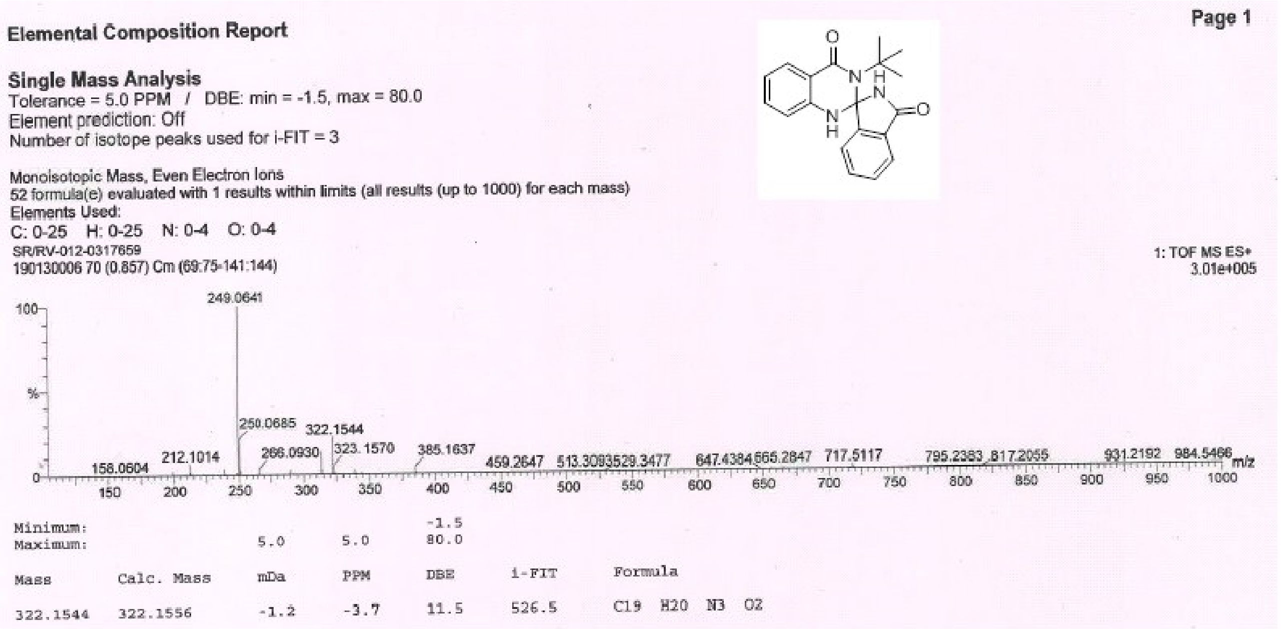

^{1}H-NMR of 3'-isobutyl-1'H-spiro[isoindoline-1,2'-quinazoline]-3,4'(3'H)-dione(1g):

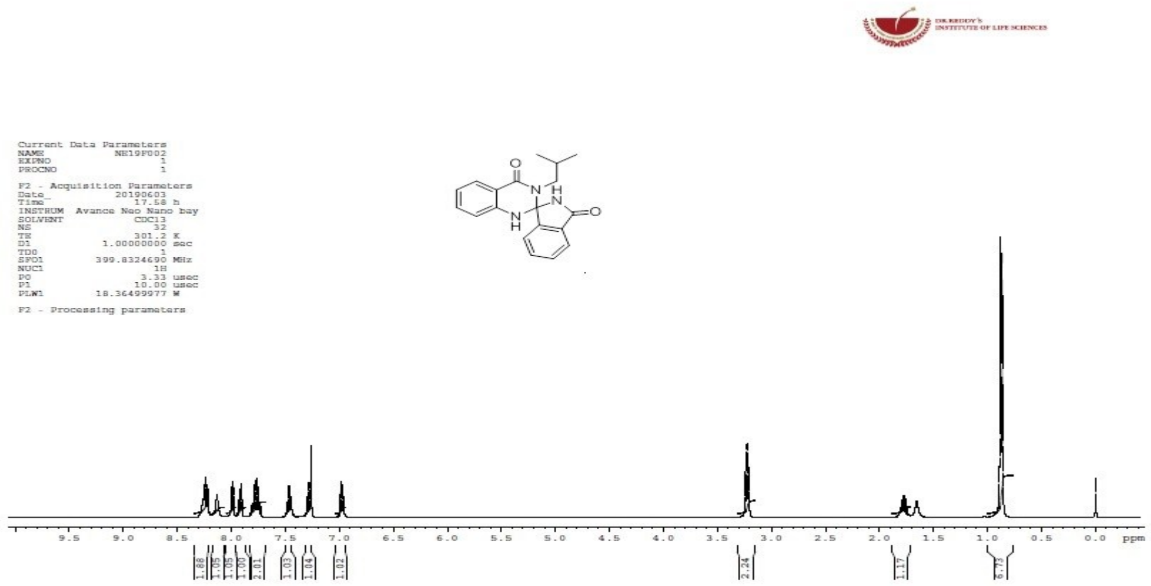

^{13}C NMR of 3'-Isobutyl-1'H-spiro[isoindoline-1,2'-quinazoline]-3,4'(3'H)-dione(1g):

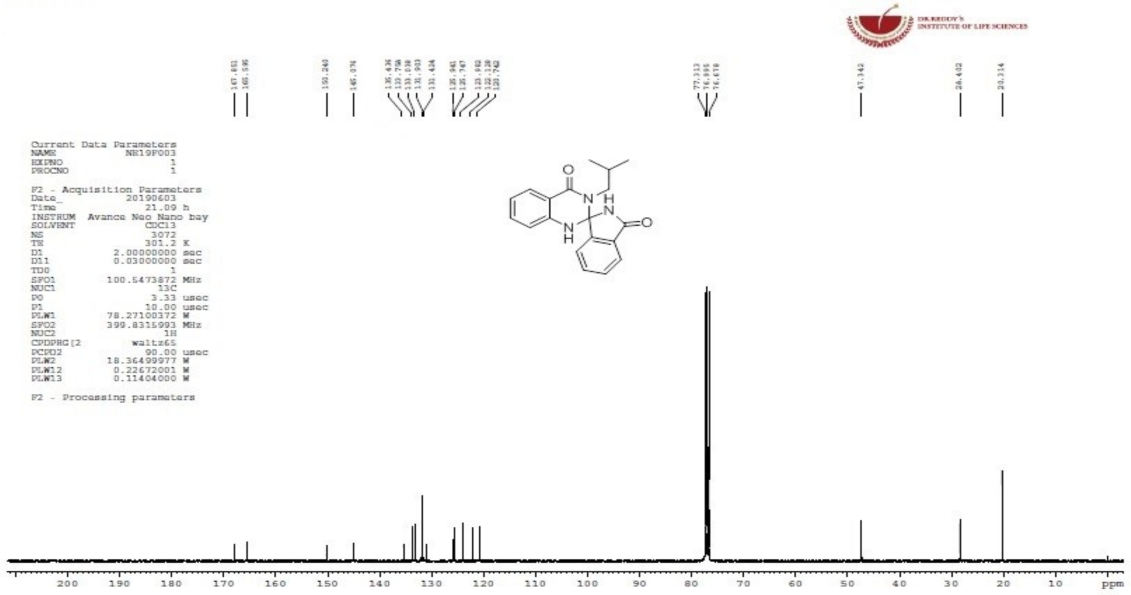

HRMS of 3'-Isobutyl-1'H-spiro[isoindoline-1,2'-quinazoline]-3,4'(3'H)-dione(1g):

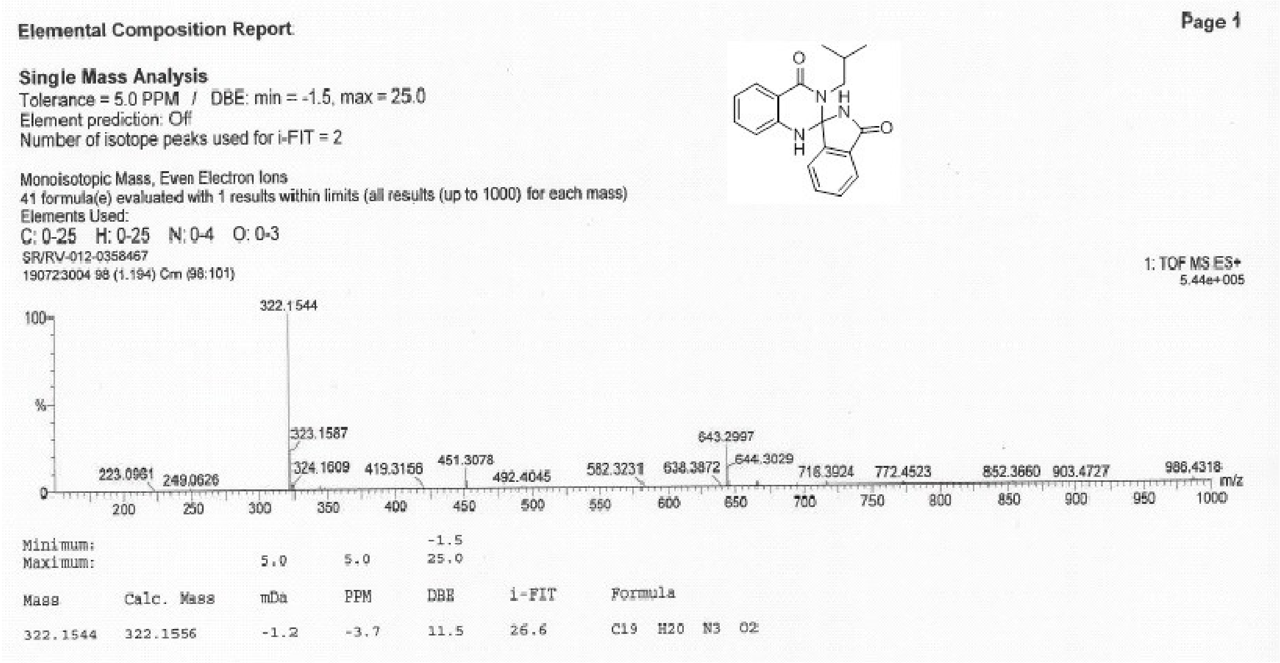

¹H-NMR of 3'-isopentyl-1'H-spiro[isoindoline-1,2'-quinazoline]-3,4'(3'H)-dione (1h):

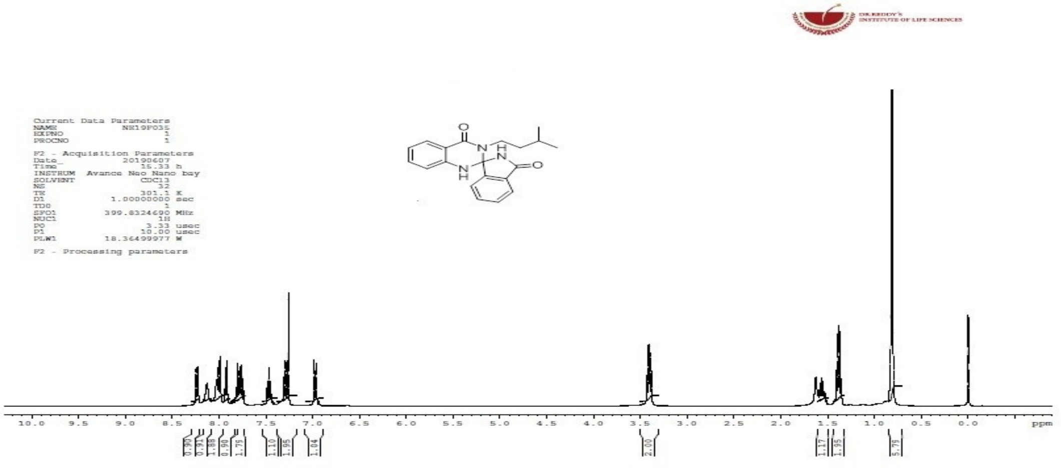

^{13}C NMR of 3'-Isopentyl-1'H-spiro[isoindoline-1,2'-quinazoline]-3,4'(3'H)-dione (1h):

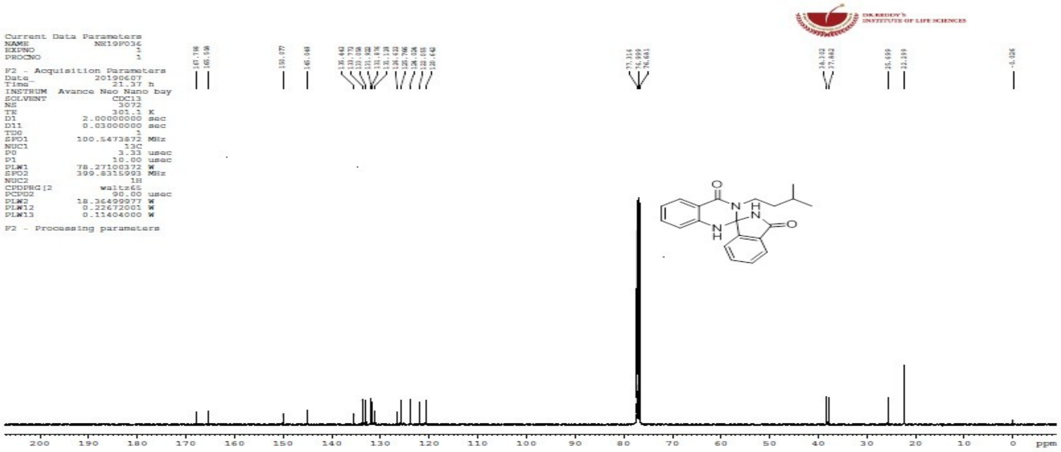

HRMS of 3'-isopentyl-1'H-spiro[isoindoline-1,2'-quinazoline]-3,4'(3'H)-dione (1h):

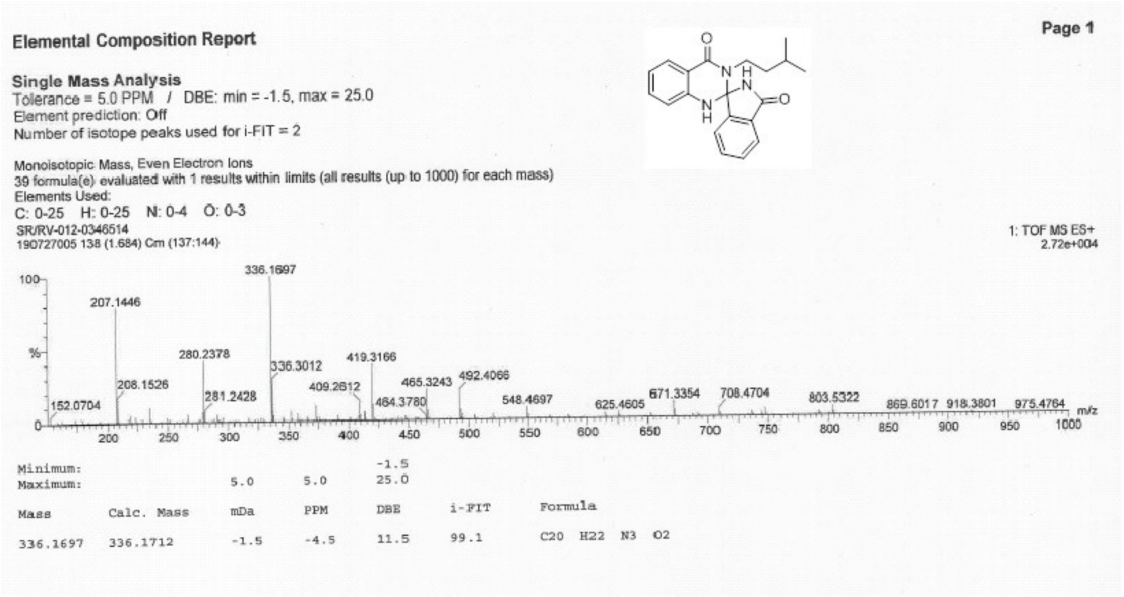

¹H NMR of 3'-Hexyl-1'H-spiro [isoindoline-1, 2'-quinazoline]-3, 4' (3'H)-dione (1i):

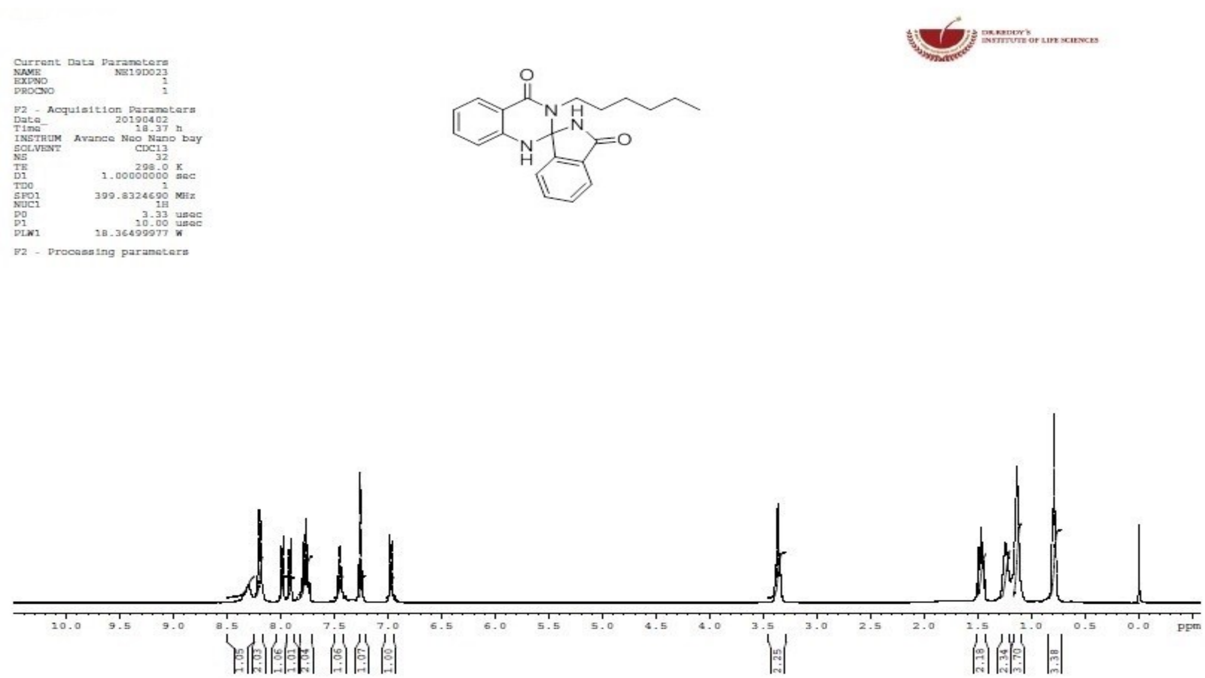

¹³C NMR of 3'-Hexyl-1'H-spiro [isoindoline-1, 2'-quinazoline]-3, 4' (3'H)-dione (1i):

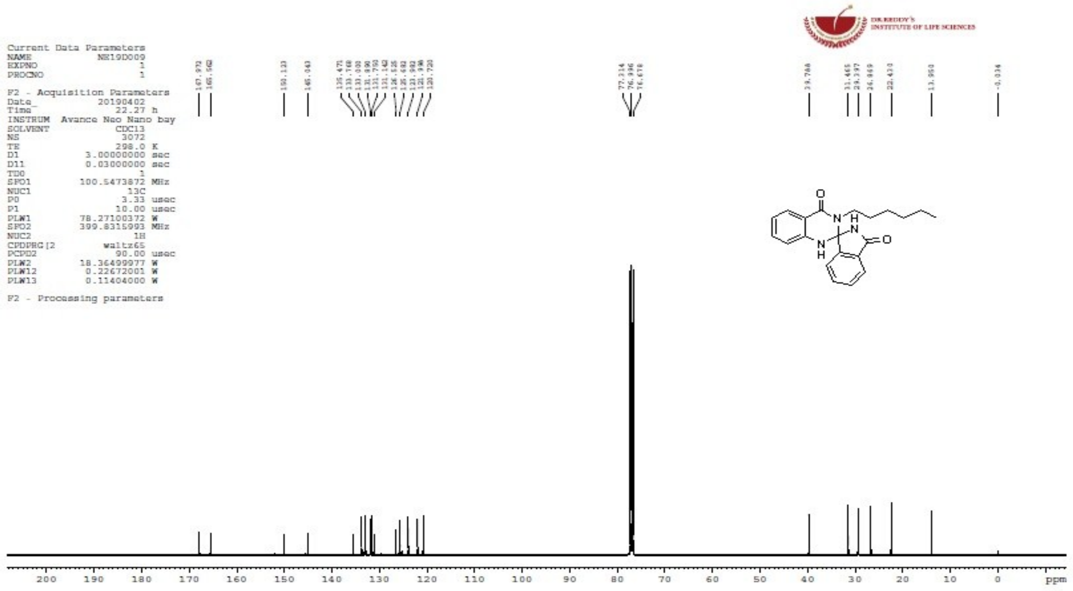

HRMS of 3'-hexyl-1'H-spiro [isoindoline-1, 2’-quinazoline]-3, 4’ (3'H)-dione (1i):

Elemental Composition Report Page 1

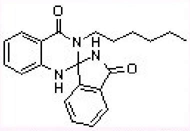

Single Mass Analysis
Tolerance = 10.0 PPM / DBE: min = -1.5, max = 50.0
Element prediction: Off
Number of isotope peaks used for i-FIT = 3

Monoisotopic Mass, Even Electron Ions
43 formula(e) evaluated with 1 results within limits (all results (up to 1000) for each mass)
Elements Used:
C: 0-25 H: 0-25 N: 0-4 O: 0-4
SR/RV-012-0300123
181127007 19 (0.435) Cm (19:22)

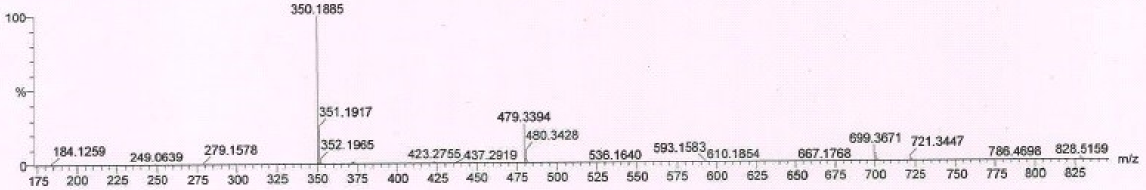

Minimum:				-1.5		
Maximum:		5.0	10.0	50.0		
Mass	Calc. Mass	mDa	PPM	DBE	i-FIT	Formula
350.1885	350.1869	1.6	4.6	11.5	86.7	C21 H24 N3 O2

^{1}H-NMR of 3'-cyclohexyl-1'H-spiro[isoindoline-1,2'-quinazoline]-3,4'(3'H)-dione (1j):

```
Current Data Parameters
NAME          NE19D024
EXPNO                1
PROCNO               1

F2 - Acquisition Parameters
Date_          20190403
Time              18.07 h
INSTRUM  Avance Neo Nano bay
SOLVENT           CDC13
NS                   32
TE                298.0 K
D1           1.00000000 sec
TD0                   1
SFO1        399.8324690 MHz
NUC1                 1H
P0                 3.33 usec
P1                10.00 usec
PLW1        18.36499977 W

F2 - Processing parameters
```

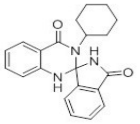

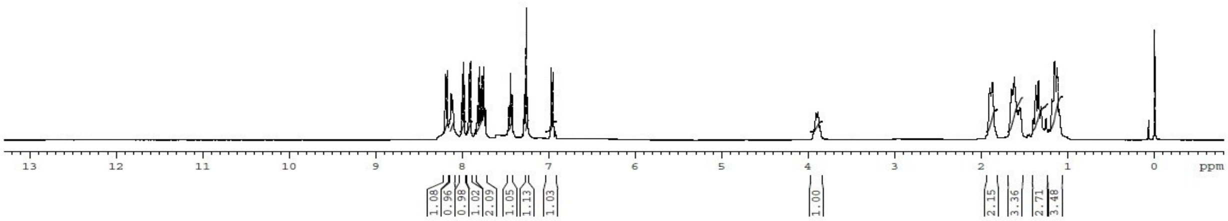

^{13}C NMR of 3'-Cyclohexyl-1'H-spiro[isoindoline-1,2'-quinazoline]-3,4'(3'H)-dione (1j):

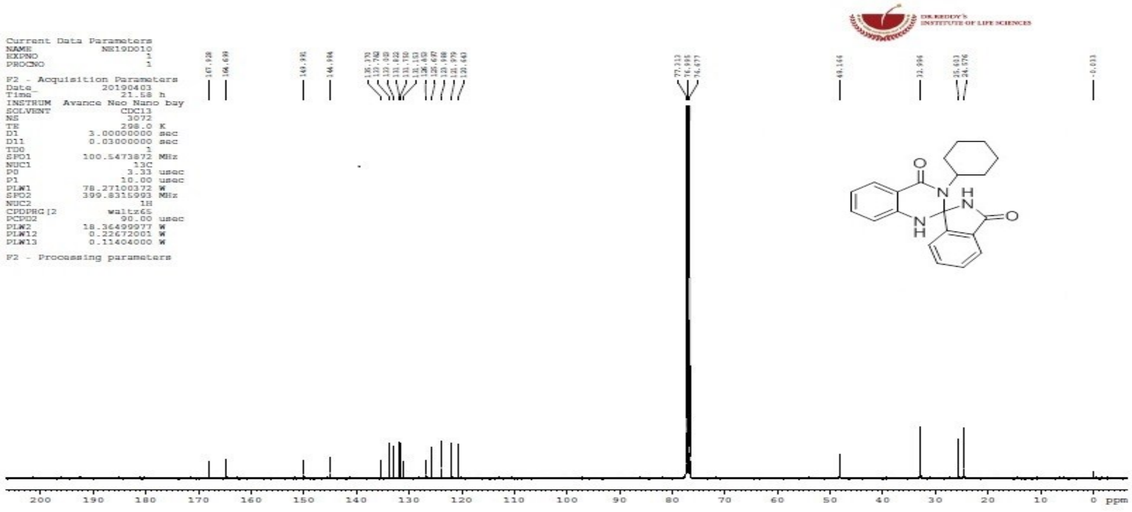

HRMS of 3'-cyclohexyl-1'H-spiro[isoindoline-1,2'-quinazoline]-3,4'(3'H)-dione (1j):

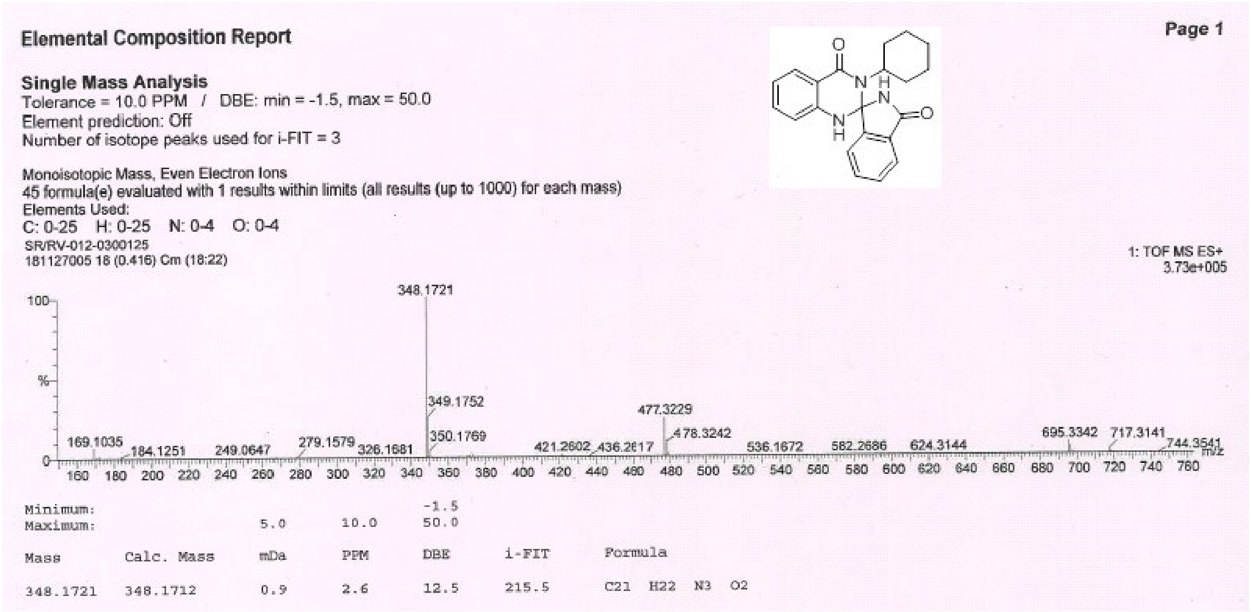

¹H-NMR of 3'-cycloheptyl-1'H-spiro[isoindoline-1,2'-quinazoline]-3,4'(3'H)-dione (1k):

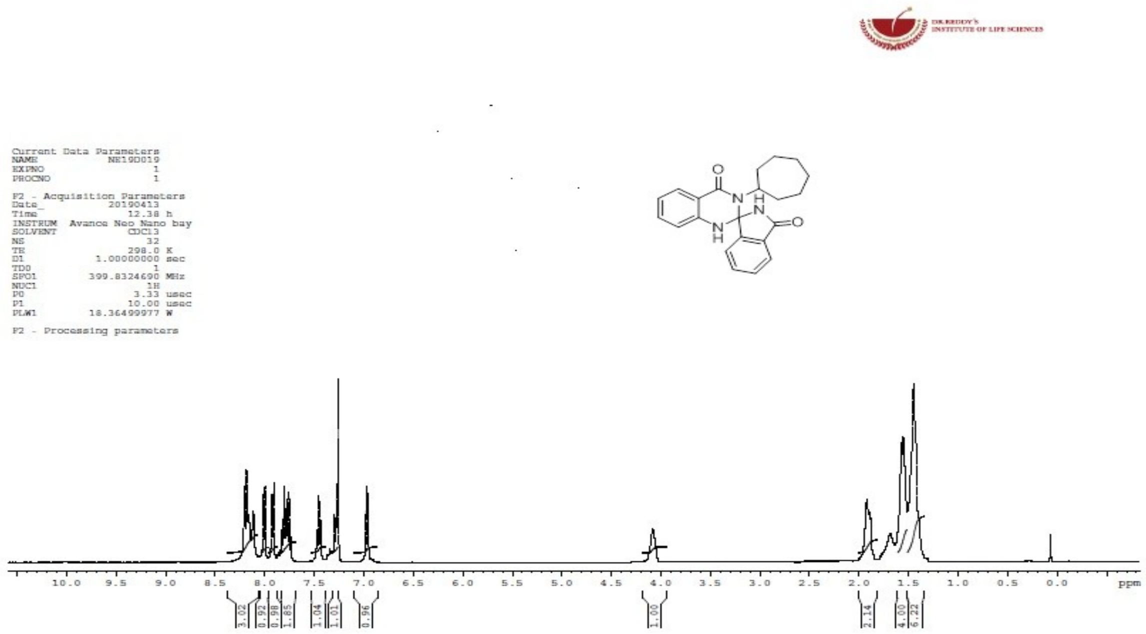

¹³C-NMR of 3'-cycloheptyl-1'H-spiro[isoindoline-1,2'-quinazoline]-3,4'(3'H)-dione (1k):

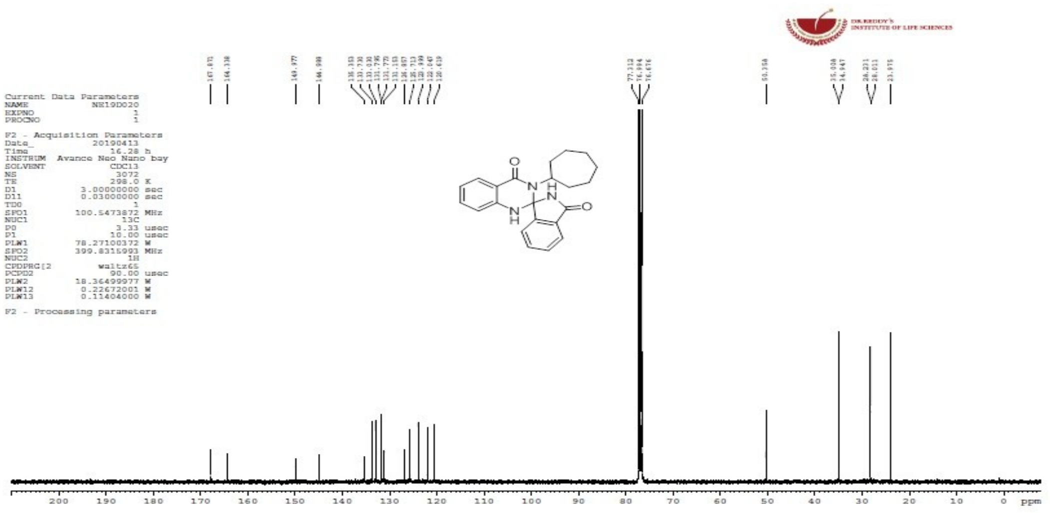

HRMS of 3'-cycloheptyl-1'H-spiro[isoindoline-1,2'-quinazoline]-3,4'(3'H)-dione (1k):

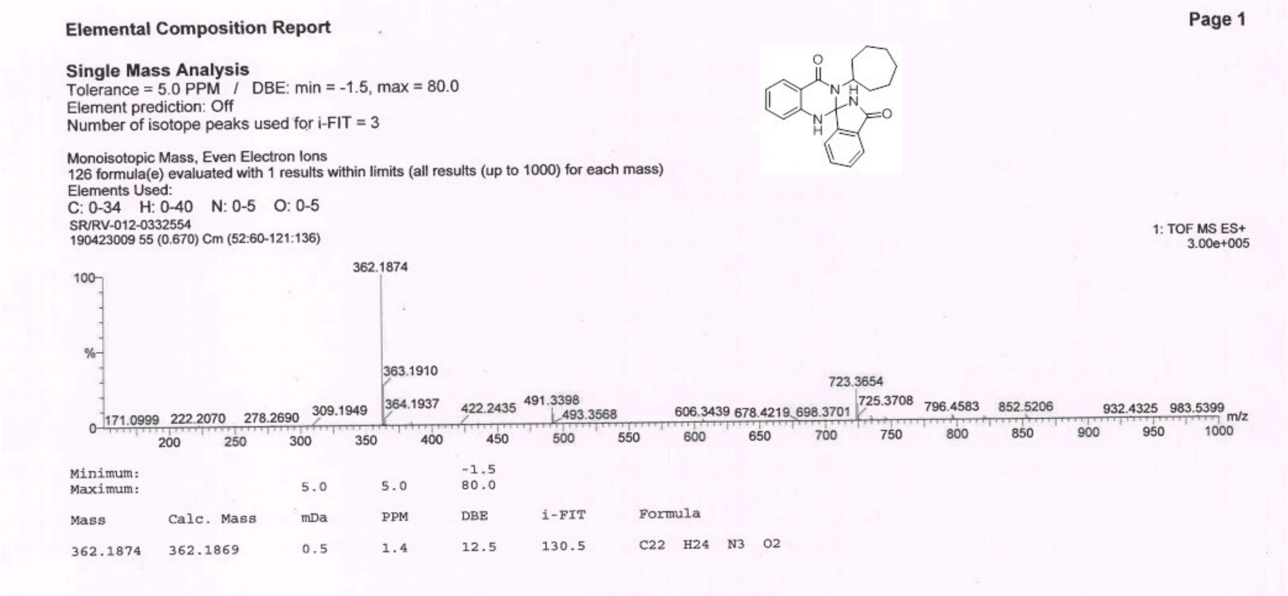

¹H-NMR of 3'-((R)-1-phenylethyl)-1'H-spiro[isoindoline-1,2'-quinazoline]-3,4'(3'H)-dione(1l):

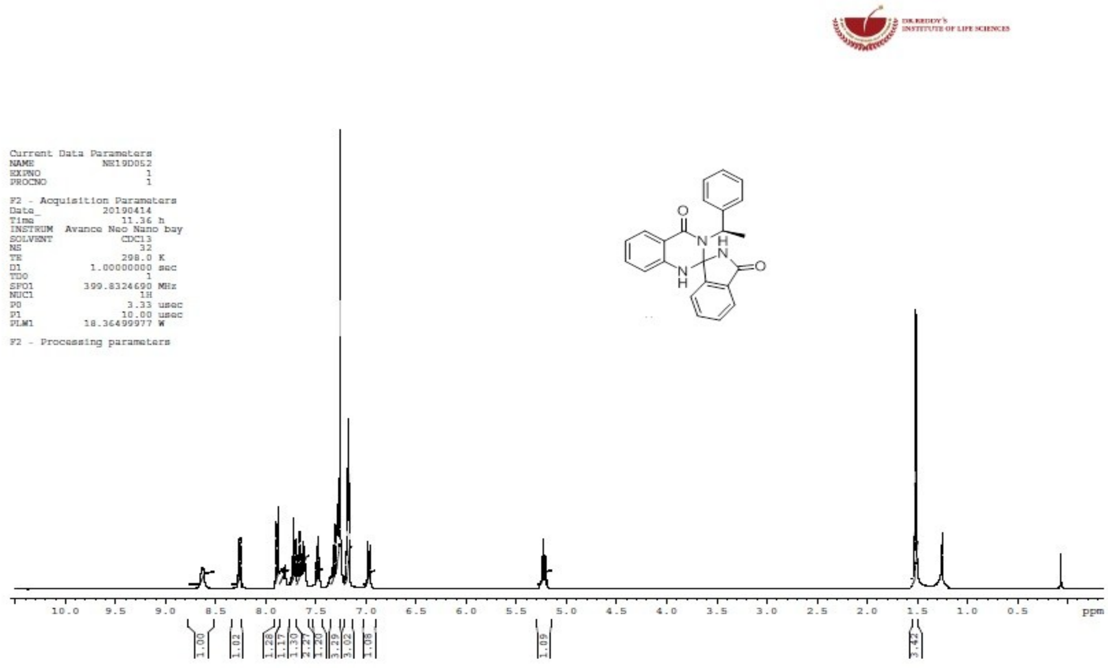

^{13}C-NMR of 3'-((R)-1-phenylethyl)-1'H-spiro[isoindoline-1,2'-quinazoline]-3,4'(3'H)-dione(1l):

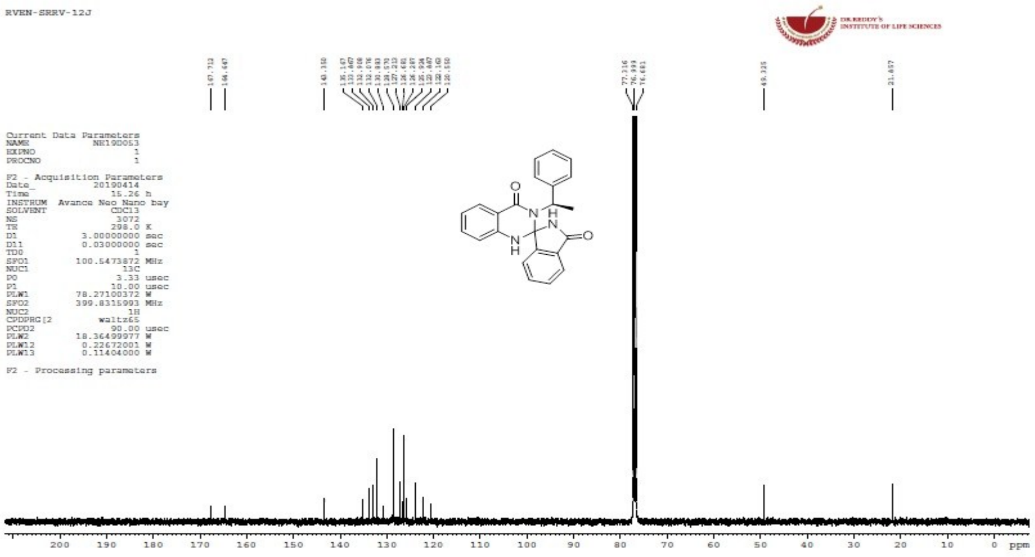

HRMS of 3'-((R)-1-phenylethyl)-1'H-spiro[isoindoline-1,2'-quinazoline]-3,4'(3'H)-dione(1l):

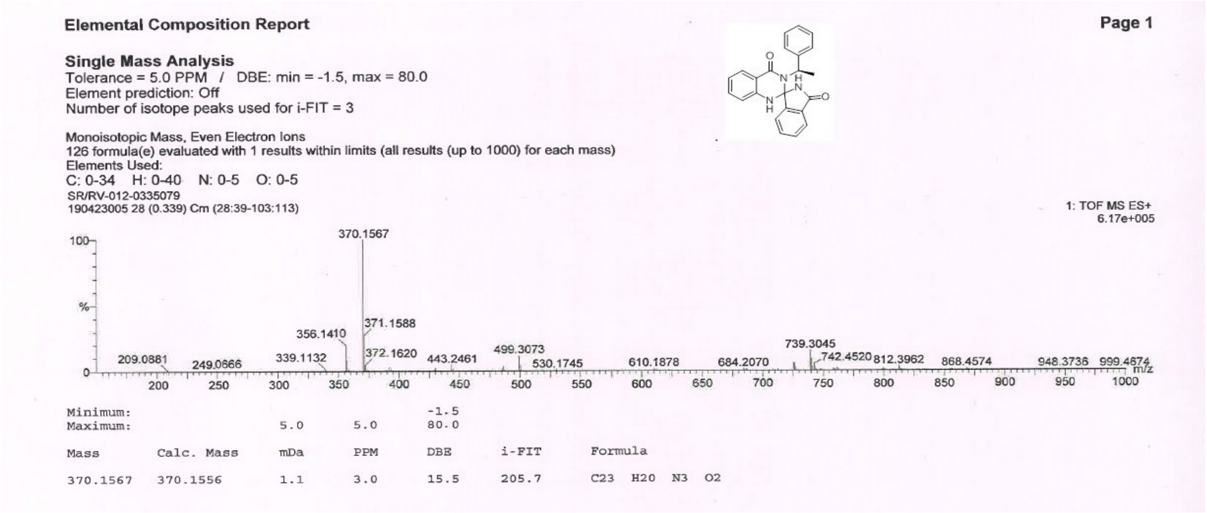

¹H-NMR of 3'-((S)-1-phenylethyl)-1'H-spiro[isoindoline-1,2'-quinazoline]-3,4'(3'H)-dione (1m):

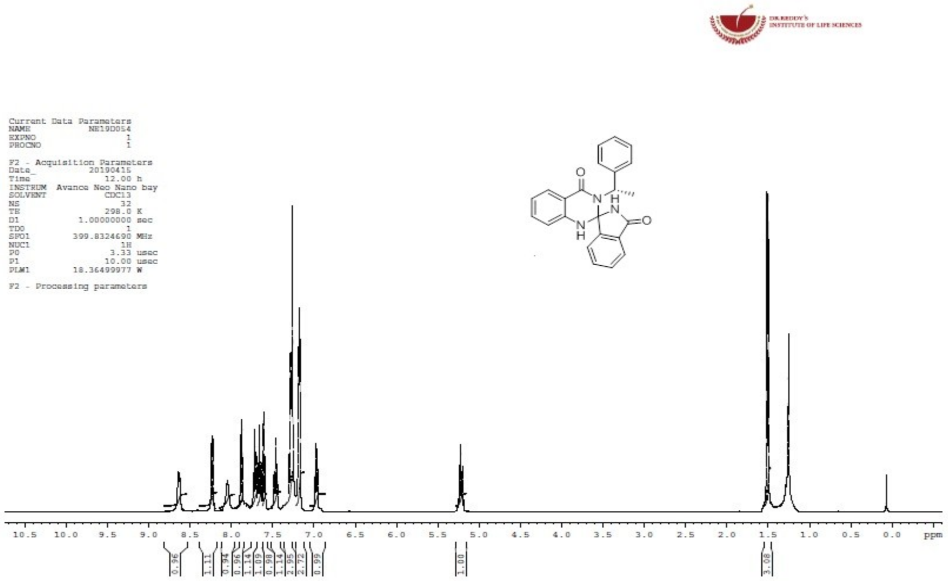

¹³C-NMR of 3'-((S)-1-phenylethyl)-1'H-spiro[isoindoline-1,2'-quinazoline]-3,4'(3'H)-dione (1m):

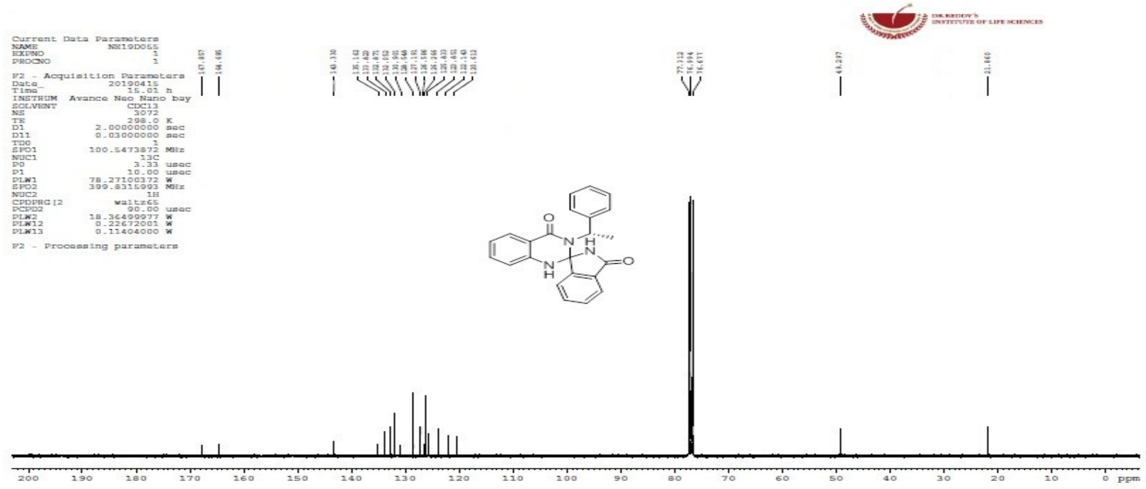

HRMS of 3'-((S)-1-phenylethyl)-1'H-spiro[isoindoline-1,2'-quinazoline]-3,4'(3'H)-dione (1m):

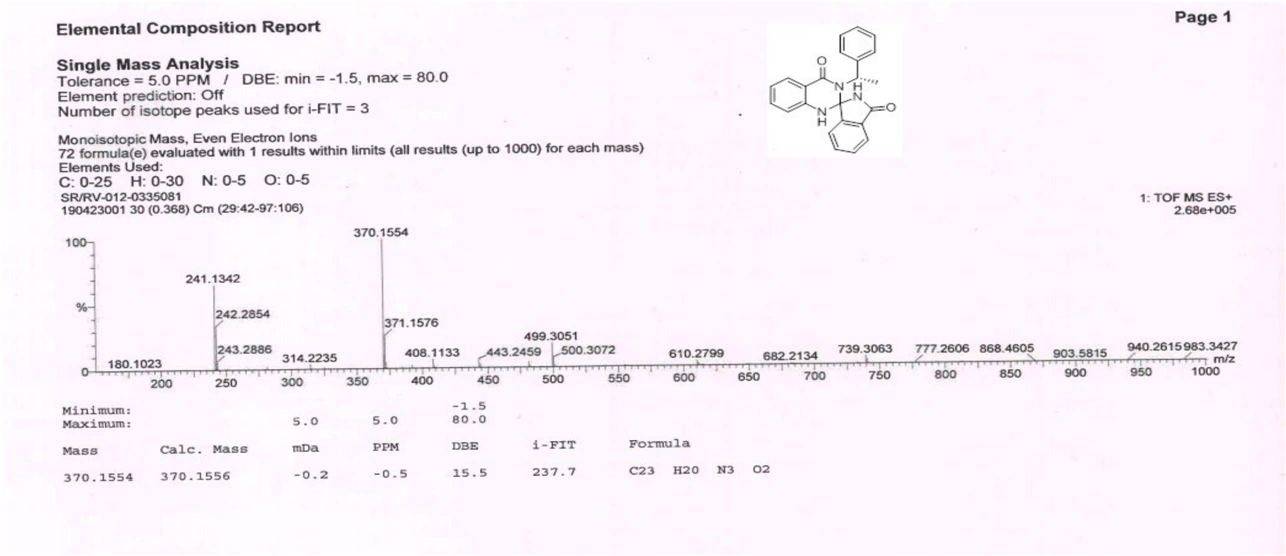

¹H NMR of 3'-((R)-1-(Naphthalen-2-yl)ethyl)-1'H-spiro[isoindoline-1,2'-quinazoline]-3,4'(3'H)-dione (1n):

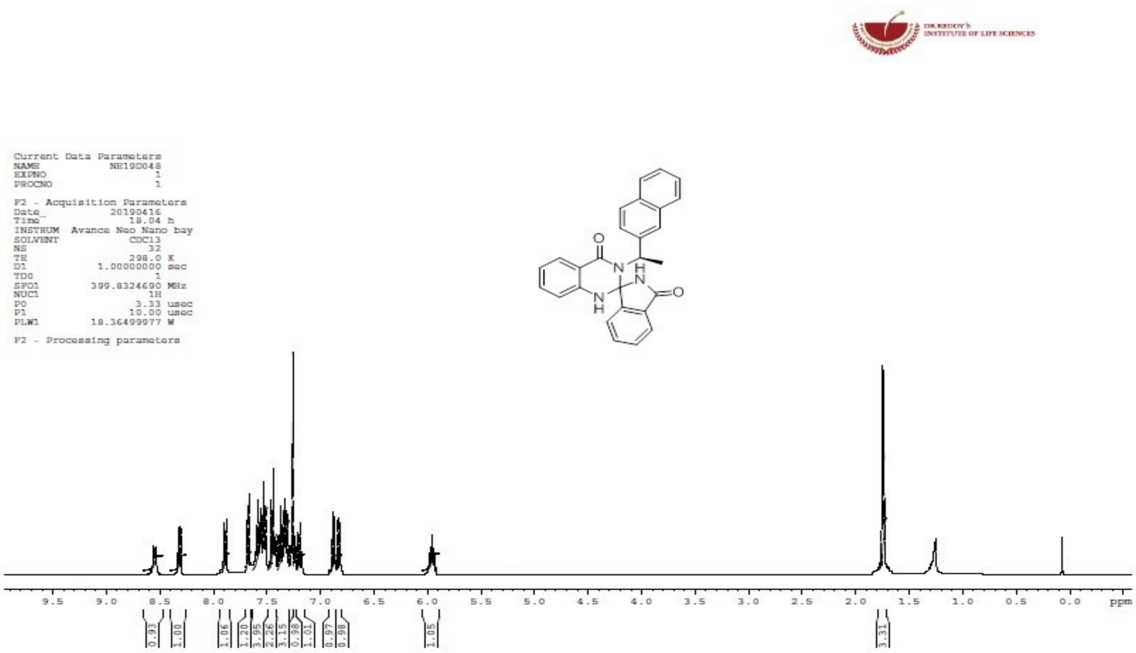

^{13}C-NMR of 3'-((R)-1-(naphthalen-2-yl) ethyl)-1'H-spiro [isoindoline-1, 2'-quinazoline]-3, 4' (3'H)-dione (1n):

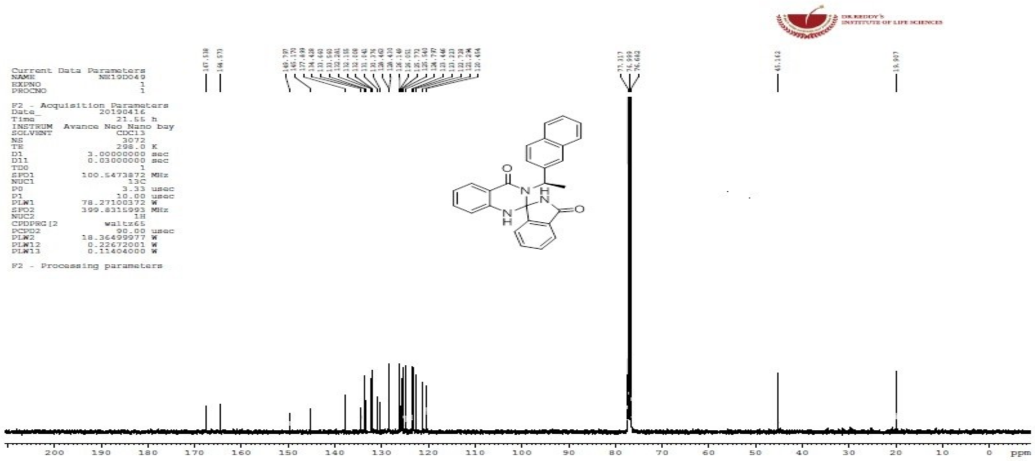

HRMS of 3'-((R)-1-(naphthalen-2-yl)ethyl)-1'H-spiro [isoindoline-1, 2'-quinazoline]-3, 4' (3'H)-dione (1n):

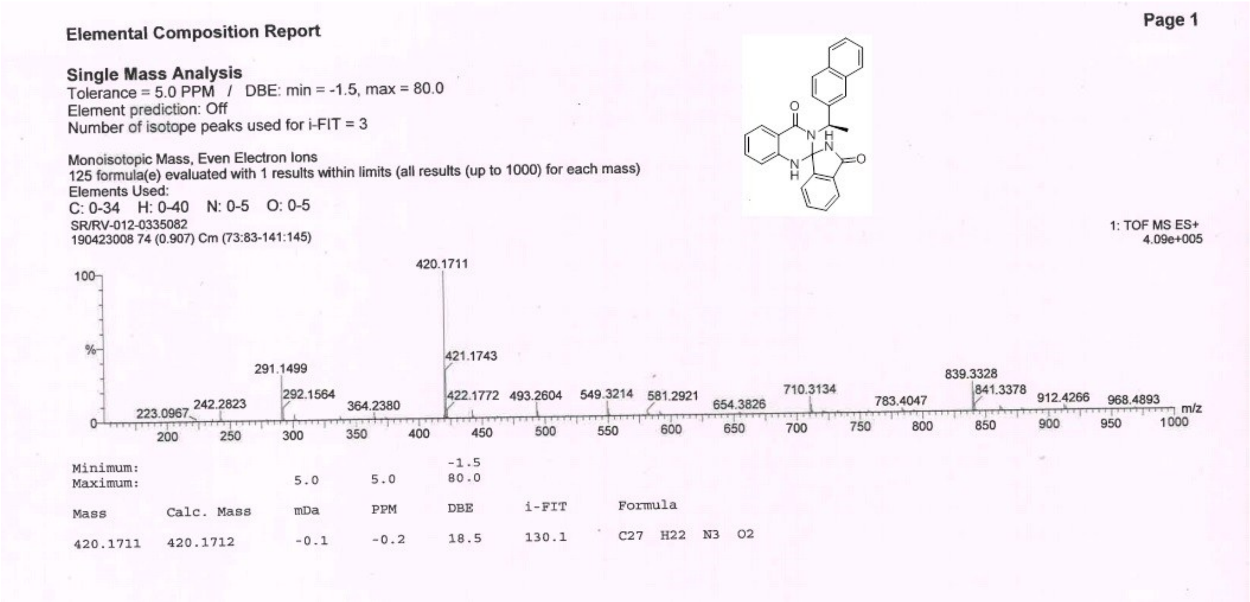

^{1}H NMR 3'-(tert-butyl)-6'-iodo-1'H-spiro[isoindoline-1,2'-quinazoline]-3,4'(3'H)-dione(1o):

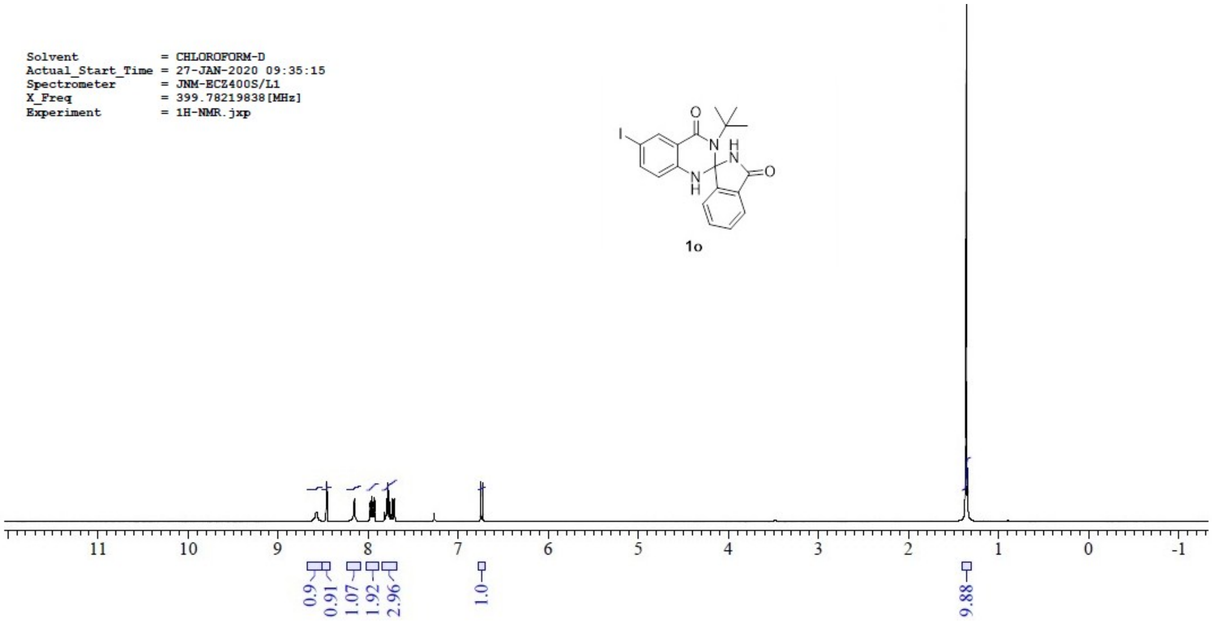

^{13}C-NMR of 3'-(tert-butyl)-6'-iodo-1'H-spiro[isoindoline-1,2'-quinazoline]-3,4'(3'H)-dione(1o):

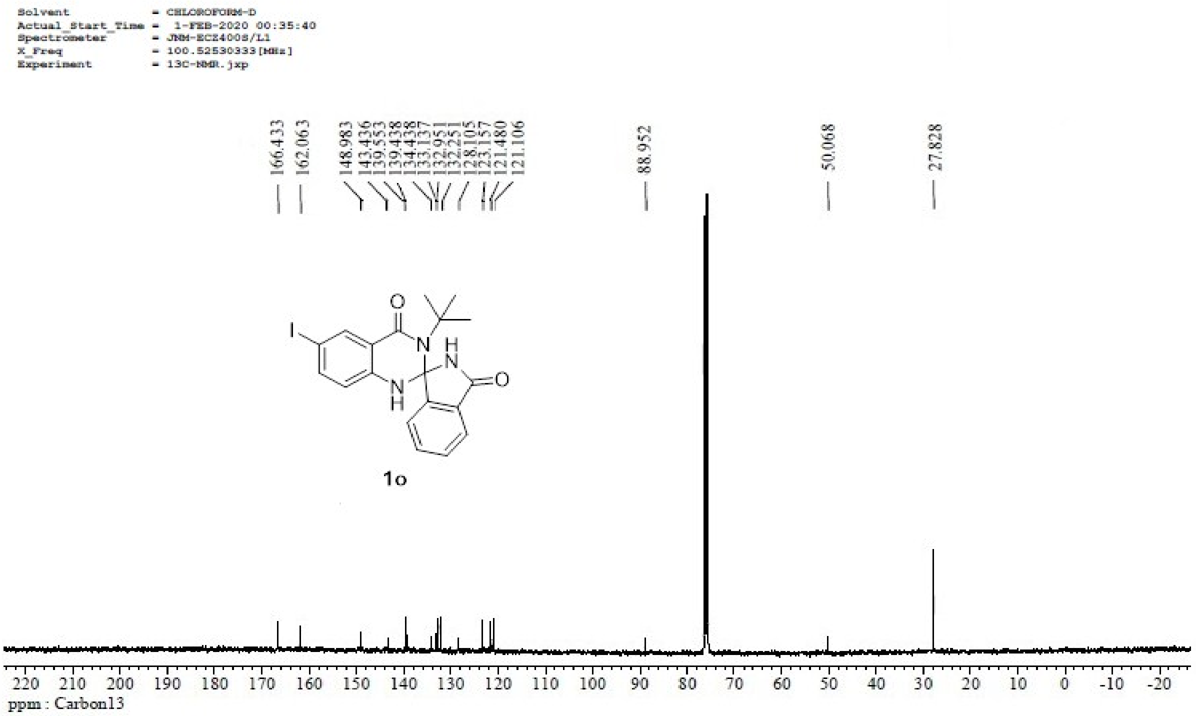

HRMS of 3'-(tert-butyl)-6'-iodo-1'H-spiro[isoindoline-1,2'-quinazoline]-3,4'(3'H)-dione(1o):

Elemental Composition Report

Page 1

Single Mass Analysis
Tolerance = 5.0 PPM / DBE: min = 1.0, max = 25.0
Element prediction: Off
Number of isotope peaks used for i-FIT = 3

Monoisotopic Mass, Even Electron Ions
66 formula(e) evaluated with 1 results within limits (all results (up to 1000) for each mass)
Elements Used:
C: 0-22 H: 0-25 N: 0-5 O: 0-3 127I: 0-1

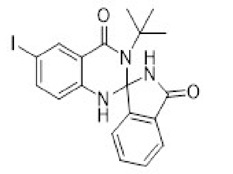

1o

SR/RV-012-0414200
200127001 33 (0.410) Cm (33)

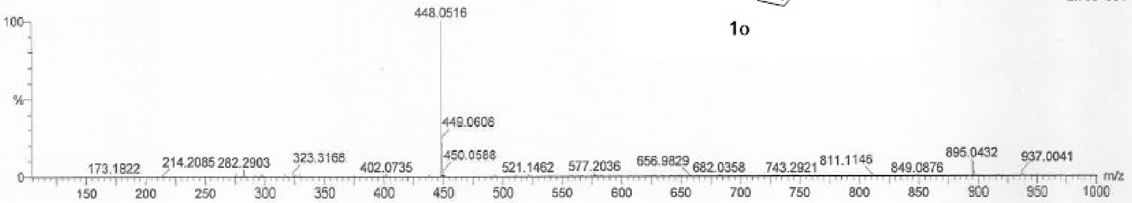

Minimum:				1.0		
Maximum:		5.0	5.0	25.0		
Mass	Calc. Mass	mDa	PPM	DBE	i-FIT	Formula
448.0516	448.0522	-0.6	-1.3	11.5	93.4	C19 H19 N3 O2 127I
